# Procleave: Predicting Protease-specific Substrate Cleavage Sites by Combining Sequence and Structural Information

**DOI:** 10.1016/j.gpb.2019.08.002

**Published:** 2020-05-12

**Authors:** Fuyi Li, Andre Leier, Quanzhong Liu, Yanan Wang, Dongxu Xiang, Tatsuya Akutsu, Geoffrey I. Webb, A. Ian Smith, Tatiana Marquez-Lago, Jian Li, Jiangning Song

**Affiliations:** 1 Biomedicine Discovery Institute and Department of Biochemistry and Molecular Biology, Monash University, Melbourne, VIC 3800, Australia; 2 Monash Centre for Data Science, Faculty of Information Technology, Monash University, Melbourne, VIC 3800, Australia; 3 School of Medicine, University of Alabama at Birmingham, Birmingham, AL 35233, USA; 4 College of Information Engineering, Northwest A&F University, Yangling 712100, China; 5 Bioinformatics Center, Institute for Chemical Research, Kyoto University, Uji, Kyoto 611-0011, Japan; 6 ARC Centre of Excellence in Advanced Molecular Imaging, Monash University, Melbourne, VIC 3800, Australia; 7 Biomedicine Discovery Institute and Department of Microbiology, Monash University, Melbourne, VIC 3800, Australia

**Keywords:** Protease, Cleavage site prediction, Machine learning, Conditional random field, Structural determinants

## Abstract

Proteases are enzymes that cleave and hydrolyse the peptide bonds between two specific amino acid residues of target substrate proteins. **Protease**-controlled proteolysis plays a key role in the degradation and recycling of proteins, which is essential for various physiological processes. Thus, solving the substrate identification problem will have important implications for the precise understanding of functions and physiological roles of proteases, as well as for therapeutic target identification and pharmaceutical applicability. Consequently, there is a great demand for bioinformatics methods that can predict novel substrate cleavage events with high accuracy by utilizing both sequence and structural information. In this study, we present Procleave, a novel bioinformatics approach for predicting protease-specific substrates and specific cleavage sites by taking into account both their sequence and 3D structural information. Structural features of known cleavage sites were represented by discrete values using a LOWESS data-smoothing optimization method, which turned out to be critical for the performance of Procleave. The optimal approximations of all structural parameter values were encoded in a **conditional random field** (CRF) computational framework, alongside sequence and chemical group-based features. Here, we demonstrate the outstanding performance of Procleave through extensive benchmarking and independent tests. Procleave is capable of correctly identifying most cleavage sites in the case study. Importantly, when applied to the human structural proteome encompassing 17,628 protein structures, Procleave suggests a number of potential novel target substrates and their corresponding cleavage sites of different proteases. Procleave is implemented as a webserver and is freely accessible at http://procleave.erc.monash.edu/.

## Introduction

Protease-specific cleavage is a ubiquitous type of irreversible post-translational modification (PTM) that occurs when proteases specifically cleave the peptide bonds between the P1 and P1′ sites of target proteins or peptide substrates [1]. Numerous experimental studies indicate that proteolytic cleavage plays a critical role in a variety of developmental and physiological processes, including cell cycle, pathway regulation, and protein degradation. On the other hand, the dysregulation of proteases is associated with numerous diseases [2]. Thus, it is very important to identify protease-specific substrate cleavage sites, as such knowledge can provide deeper insights into the mechanisms and biological functions of proteases, which in turn might lead to novel therapeutic targets and pharmaceutical applicability. However, current existing experimental methods for protease substrate cleavage site identification are expensive, labour-intensive, and time-consuming. Therefore, the development of cost-effective computational approaches for precise prediction of protease-specific proteolytic events is very important. Such tools can not only provide high-quality predictions of target substrates for a specific protease, but also guide hypothesis-driven experimental efforts to identify substrate specificity and associated biological functions of proteases.

Due to the importance and the benefits of computational predictions of protease-specific target substrates, over the past two decades, more than 20 computational methods have been proposed [3], [4]. In our recent review paper, we categorized these methods into two major groups according to the employed methodologies: (i) sequence-scoring function-based methods, such as PoPS [5], SitePrediction [6], and CAT3 [7], and (ii) machine learning methods, such as Pripper [8], Cascleave [9], PROSPER [10], LabCaS [11], ScreenCap3 [12], Cascleave 2 [13], iProt-Sub [14], and PROSPERous [15]. These publicly available computational tools have successfully guided experiments in finding novel cleavage sites and obtaining a better understanding of protease–substrate interactions.

A number of encouraging studies have been done regarding the development of computational methods and tools for predicting protease-specific cleavage sites. However, all of these existing prediction methods are developed based on protein sequences and they are only used for predicting the cleavage sites from substrate sequences. Previous studies have shown that protease cleavage sites are primarily distributed in loop regions of the substrate proteins, while cleavage within other structural regions of substrate proteins, such as α-helices and β-sheets, is also possible [16], [17], [18]. These findings indicate protease substrate cleavage specificity at the secondary structure (SS) level. The majority of existing predictors did not consider the structure-level preference and parameters, which can potentially improve the prediction performance and also help better understand the biological functions of proteases.

In this study, we introduce Procleave to fill the knowledge gap outlined above and enhance protease substrate cleavage site prediction by incorporating 3D structural features of substrate cleavage segments. More specifically, Procleave uses the data curated from the MEROPS database [19] and maps substrate sequences to PDB structures by performing BLAST search, thereby generating an extensive 3D structural substrate dataset. Multi-faceted sequence and structural features are then extracted, which are further integrated into a novel conditional random field (CRF) algorithm with a data-smoothing framework to train cleavage site prediction models. A comprehensive performance test confirms that smoothed structural features combined with sequence-based features can greatly improve the prediction performance. Subsequently, we implement a webserver for 27 major proteases, taking advantage of the findings in this study, and make it publicly accessible.

## Method

### Overall framework

**Figure 1:**
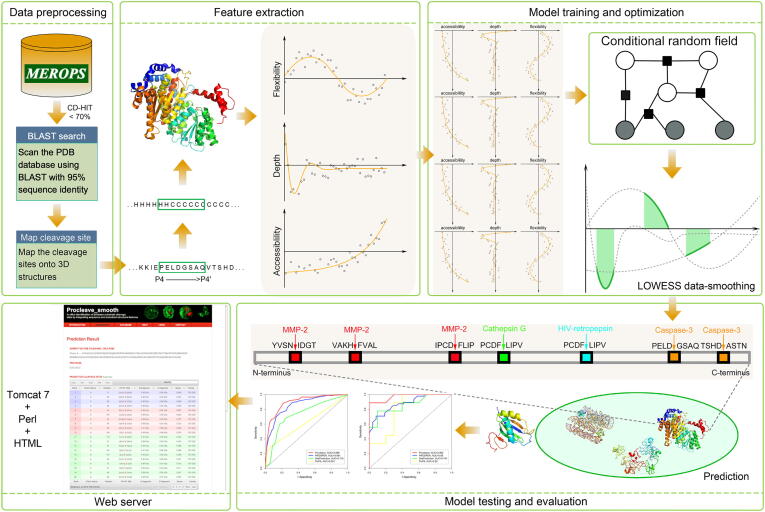
**The overall framework of Procleave** There are five major steps in the framework of Procleave, including data pre-processing, feature extraction, model training and optimization, model testing and evaluation, as well as web server development.

Figure 1 provides an overview of the Procleave framework. Five major steps are involved in the construction and evaluation of Procleave. At the first step, *i.e.*, data collection and pre-processing, the benchmark training and independent test datasets were collected from MEROPS [19]. At the second step, multi-faceted sequence features and 3D structure features were generated. At the third step, a novel integrative CRF framework was developed for model training and optimization. At the fourth step, the trained CRF models were further evaluated and validated by performing the independent test. A performance comparison with currently existing methods was also conducted. At the final step, the Procleave webserver was implemented to facilitate public use.

### Dataset collection and pre-processing

The experimentally verified protein substrate cleavage annotations for training and benchmarking Procleave were extracted from the MEROPS database (Release 9.0) [19]. MEROPS is a public resource and knowledgebase for experimentally validated protease substrates and cleavage sites, which is accessible via https://www.ebi.ac.uk/merops/. To develop reliable prediction models and objectively evaluation the model performance, we discarded highly homologous sequences from the initial substrate dataset with a sequence identity (SI) threshold of 70% between any two substrate protein sequences. This avoids overestimating the prediction performance in cross-validation tests. It is noticeable that a number of existing studies used SI cut-off values of 70% [9], [14], [15] or a higher, *e.g.*, 80% [12]. The MEROPS database was recently updated (Release 12.0, 26-April-2019) and we decided to use all the newly added protease substrates and cleavage sites as the independent test dataset to assess the performance of trained Procleave models and conduct the performance comparison with existing methods. In addition, in order to perform a more fairly independent test, we used a stricter SI threshold (30%) to remove the sequence redundancy in the independent test dataset. CD-HIT [20] was applied to remove the redundant sequences between the independent test datasets and training datasets at the SI threshold of 30%. This ensures that any two substrates in the training and independent test datasets have a SI of <30%. A statistical summary of both benchmark and independent test datasets is provided in Tables S1 and S2, respectively. Subsequently, the remaining sequences were mapped to PDB [21] by performing PSI-BLAST [22] to search against the PDB sequence database (using the ‘pdbaa’ file) with three iterations, with an e-value of 10^−3^, and a SI threshold of 95%. We only retained the X-ray crystallography (X-ray) structures, while nuclear magnetic resonance (NMR) and electron microscopy (EM) structures were discarded. After this procedure, all substrate cleavage sites were mapped onto respective 3D structures using our in-house Perl script and all of these cleavage sites were used as positive samples to train the Procleave models. Sites that have been not annotated as cleavage sites in substrate proteins were considered as negative samples. Accordingly the same number of negative sites was randomly selected as that of the positive samples. In this study, a gallery of all mapped respective 3D structures with visualized cleavage sites can be accessed at http://procleave.erc.monash.edu/gallery.html.

### Feature engineering

The substrate cleavage site prediction task can be regarded as a binary classification problem. Each cleavage site is denoted as an *N*-dimensional feature vector *F* = {*f*_1_, *f*_2_, …, *f****_N_***}. Three major types of features were extracted, namely structural features, sequence features, and chemical group features. A detailed description of each feature type is presented below.

#### Structural features

In this study, several different types of 3D structural descriptors were extracted from the P4–P4′ local windows surrounding cleavage sites, which include:

(1) Protrusion and depth index. We calculated the protrusion (cx) index and the depth index by CX [23] and DPX [24] programs, respectively.
(2) Solvent accessibility. Naccess [25] was employed to compute the absolute and relative solvent accessibility features using the default settings. There are five types of solvent accessibility features, including all atoms, total side chain, main chain, non-polar side chain, and all-polar side chain solvent accessibility.
(3) Packing. Packing was calculated using the method proposed previously [26].
(4) Molecular surface accessibility. Molecular surfaces are either solvent-accessible surfaces (SAS) or solvent-excluded surfaces (SES). Both were calculated by the MSMS program [27].
(5) Secondary structure features. The DSSP program [28] was used to calculate the secondary structure features. These encompass hydrogen bonds, secondary structures (eight classes were transformed to three classes, *i.e.*, α-helix, β-sheet, and coil), and backbone torsion angles. The HBPLUS v.3.06 program [29] was used to calculate the hydrogen bond.
(6) Solvent exposure properties. Half-sphere exposure properties were also used as candidate features. They were extracted using the Biopython package [30]. They included contact number (CN), the number of Cα atoms in the upper half-sphere (HSEAU), the number of Cα atoms in the lower half-sphere (HSEAD), the number of Cβ atoms in the upper half-sphere (HSEBU), and the number of Cβ atoms in the lower half-sphere (HSEBD).
(7) B-factor. The B-factor values of all atoms were extracted from PDB files, with the average values being used as the input feature [31].

#### Sequence features

We employed the binary encoding scheme to extract and encode sequence features. In particular, a sliding window approach (P4–P4′) centred around the potential cleavage sites was used to extract the local sequence features. Each amino acid (AA) residue was encoded by a binary vector with 20 dimensions. Therefore, the total number of dimensions of the obtained vector is 8 × 20 = 160.

#### Chemical group features

Apart from structural and sequence features, the chemical/structural groupings of AAs were also used as candidate features. According to the chemical/structural properties, 20 AAs were clustered into eight chemical groups [32]. These include sulfur-containing (residues C and M), aliphatic 1 (residues A, G, and P), aliphatic 2 (residues I, L, and V), acidic (residues D and E), basic (residues H, K, and R), aromatic (residues F, W, and Y), amide (residues N and Q), and small hydroxy (residues S and T) residues. Then, these eight chemical groups were encoded as input features using the one-hot encoding. The total number of dimensions of the chemical group features is 8 × 8 = 64 (for any 8-AA window).

### Model training and optimization

#### CRFs and LOWESS data smoothing

CRFs are a type of undirected graphical models originally introduced by Lafferty et al. [33] to deal with the segmentation and labelling tasks of text sequences. CRFs have been proven to be effective in a number of applications with structured outputs, such as information extraction, image processing, and parsing. A CRF is an undirected graph, and its nodes can be categorized as two disjoint sets, namely the observed variables X and the output variables Y. Its principle is to define a conditional probability distribution p(Y|X) over label sequences $Y=\{y_{1},y_{2},\cdots ,y_{n}\}$, given the observational sequence $X=\{x_{1},x_{2},\cdots ,x_{n}\}$. Yis a sequence of hidden state variables that needs to be inferred given the observation. $y_{1},\cdots ,y_{i},y_{i+1},\cdots ,y_{n}$ are structured to form a chain, with an edge between each $y_{i}$ and $y_{i+1}$. The distribution of the network has the following form:

(1) $$p\left(Y|X\right)=\frac{1}{Z(X)}\exp\left(\sum_{k=1}^{K}\lambda_{k}f_{k}\left(y_{i},y_{i-1},x_{i}\right)\right),$$

where $Z\left(X\right)=\sum_{y_{i}}exp\left(\sum_{k=1}^{K}\lambda_{k}f_{k}\left(y_{i},y_{i-1},x_{i}\right)\right)$, *K* denotes the number of class labels (*e.g.*, *K* = 2 stands for a two-class classification), $\lambda_{k}$ is the weight vector of features, and $f_{k}$ is the function of features for the clique $\left\{y_{i},y_{i-1},x_{i}\right\}$.

Since a CRF does not have the assumption for the distribution of inputs and, instead, finds the decision boundary directly, it may be considered as an extended version of logistic regression to model sequential data. CRFs have been applied to bioinformatics rather recently and have delivered promising results, such as for gene prediction [34] and phosphorylation sites prediction [32]. CRFs can capture sophisticated dependencies and combine information from different aspects. The specific advantages of CRFs are well-suited for incorporating structural information into a cleavage site prediction algorithm. Many of the structural parameters are closely related, and structural parameters contain important information for determining the potential cleavage site that might be better captured by CRFs.

In this study, our input variables *X* are the structural, sequence, and chemical group features of a given substrate peptide and the output variables are binary labels corresponding to “cleavage site” or “non-cleavage site”. The CRF models were trained by maximizing the likelihood that the positive samples of a training set were cleavage sites, given their structural, sequence, and chemical group features. We used the open source package CRF++ (version 0.54) and, as part of the CRF implementation, used Boolean feature functions to train the models. As the Boolean feature functions evaluate one of the two states of being true or false for a feature appearing at an exact position, all structural features are regarded in the form of discrete instead of continuous values during the model training. In addition, considering that the substrate cleavage depends on the overall 3D shape or neighbourhood of multiple AAs, structural features recognized by cleavage sites, *e.g.*, the overall shape of the P4–P4′ segment surrounding the potential cleavage sites, we combined CRF with a LOWESS data-smoothing approach [35] and examined whether cleavage site prediction could be further improved. Specifically, feature optimization first ran the LOWESS smoothing algorithm on the input vectors of each structural feature. Then the resulting vectors were discretized into equally sized bins to group similar values for use by the Boolean feature functions. Algorithm 1 describes the detailed procedures of the LOWESS smoothing algorithm.

**Table:** 

**Algorithm 1 LOWESS data-smoothing algorithm**
**Input:**
Range value, $range; Initial feature array, @iniArry;
**Output:**
Smoothed feature array, @smoothedArray;
1: **for** each $i∈[1,$#iniArry]**do**
2: $avey=$avex=$norm=$weight=0;
3: **if**${Expression}_{N}$
4: **for** each $\$j\in [S_{N}]$**do**
5: **calculate**$weight;
6: $\$avey+=\$weight\times \$iniArray\left[\$j\right];$
7: $avex+=$weight×$j;
8: $norm+=$weight;
9: **end for**;
10: $avey=$avey/$norm;$avex=$avex/$norm;
11: $mtop=$mbot=0;
12: **for** each $\$k\in [S_{N}]$**do**
13: $\$weight={\left(1-\left|{\left(\left(\$i-\$k)/(2\times \$range-\$i+1\right)\right)}^{3}\right|\right)}^{3}$;
14: $\$mtop+=\$weight\times (\left(\$k-\$avex\right)\times \left(\$array\left[\$k\right]-\$avey\right))$;
15: $\$mbot+=\$weight\times ({\left(\$k-\$avex\right)}^{2})$;
16: **end for**;
17: $\$smoothedArray\left[\$i\right]=\left(\frac{\$mtop}{\$mbot}\right)\times \$i+\left(\$avey-\left(\frac{\$mtop}{\$mbot}\right)\times \$avex\right);$
18: **end if**;
19: **end for**;

The input to Algorithm 1 was the smoothing range $range and the initial feature vector @iniArry, which needed to be smoothed and tuned. In this study, each type of structural feature was described by an 8-bit vector, where each bit was associated with the feature value of a local sliding window (P4–P4′) surrounding the potential cleavage site. The output of Algorithm 1 was the 8-bit vector $smoothedArray. The smoothing procedure was performed in a ‘for’ loop. At step 1, $#iniArray was the length of feature vector, which equals to eight. At the second step, four variables, namely $avey, $avex, $norm, and $weight, were set to 0. These variables represented the average value of *y* (*i.e.*, values of the features), the average value of *x* (positions of the feature vector), the normalization variable, and the weight of the variable, respectively. At step 3, the if statement has three different expressions ${\mathrm{Expression}}_{N}$, which can be presented as:

**Table:** 

${Expression}_{1}$	$i-$range<0;
${Expression}_{2}$	$range+$i>$#iniArray;
${Expression}_{3}$	Others.

For these three expressions, the range $S_{N}$ of the parameter $range in the step 4 and step 12 is different:

**Table:** 

${Expression}_{1}$	$S_{1}$	[1,2×$range];
${Expression}_{2}$	$S_{2}$	[$#iniArray-2×$range,$#iniArray];
${Expression}_{3}$	$S_{3}$	[$i-$range,$i+$range].

Then, at step 5, the weight of the variable was calculated. The method used for calculating the variable weight is also different:

**Table:** 

$S_{1}$	$\$weight={\left(1-\left|{\left(\left(\$i-\$j)/(2\times \$range-\$i+1\right)\right)}^{3}\right|\right)}^{3}$;
$S_{2}$	${\$weight=\left(1-\left|{\left(\left(\$i-\$j)/(\$x-\$\#iniArry-2\times \$range+1\right)\right)}^{3}\right|\right)}^{3};$
$S_{3}$	${\$weight=\left(1-\left|{\left(\left(\$i-\$j)/(2\times \$range+1\right)\right)}^{3}\right|\right)}^{3}$.

At steps 6 and 7, $weight was used to calculate the normalized values of x and y. Then, $avey and $avex were updated at step 10 by dividing the normalization variable calculated at step 8. At step 11, the smoothed value of $mtop and the smoothed bottom value of $mbot were initialized to 0. At steps 12–16, these two variables were calculated and updated, and at step 17 the final output $smoothedArray was generated according to these two values.

We set the smoothing range $range from 1 to 5 and the bin number from 1 to 10, respectively, in this study. The smoothing procedure and the number of bins for each type of structural feature were optimized by maximizing the area under the curve (AUC) of the receiver operating characteristic (ROC) curves on the 5-fold cross validation test using the benchmark dataset. In this way, by optimizing the smoothing range and the number of bins for each of the structural features, the optimal combination of smoothing and discretization that best represented structural features of all samples in the training set could be determined.

### Performance evaluation

To assess the performance of the Procleave models and benchmark it with other currently available methods, a set of five commonly used performance measures were applied, including sensitivity (Sn), specificity (Sp), precision, accuracy (Acc), Matthew’s correlation coefficient (MCC), and AUC. Sn, Sp, Precision, Acc, and MCC are defined as:

$$Sn=\frac{\mathrm{TP}}{\mathrm{TP}+FN}$$

$$Sp=\frac{\mathrm{TN}}{\mathrm{TN}+FP}$$

$$Precision=\frac{\mathrm{TP}}{\mathrm{TP}+FP}$$

$$Acc=\frac{\mathrm{TP}+TN}{\mathrm{TP}+TN+FP+FN}$$

$$MCC=\frac{\mathrm{TP}\times TN-FP\times FN}{\sqrt{\left(TP+FP\right)\times \left(TP+FN\right)\times \left(TN+FP\right)\times \left(TN+FN\right)}}$$

where TP, TN, FP, and FN represent the numbers of true positives, true negatives, false positives, and false negatives, respectively. Moreover, we plotted the ROC curves and accordingly calculated the AUCs, as a primary measure to assess the prediction performance of Procleave models and all compared methods.

## Results and discussion

### Characterization of structural features in the proximity of cleavage sites

**Figure 2:**
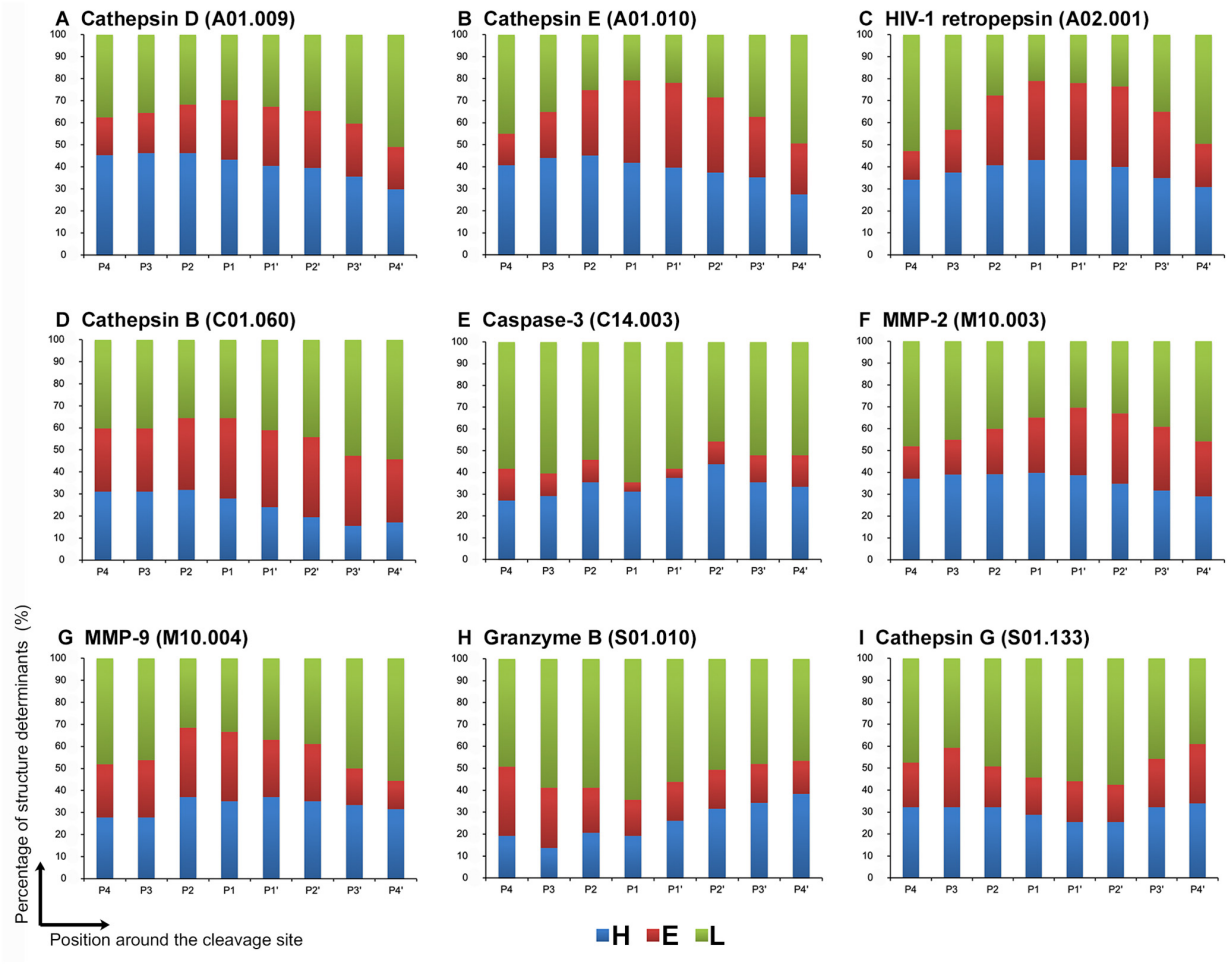
**Structural determinants of the substrate specificity of nine proteases across the P4**–**P4′ cleavage sites** **A.** Cathepsin D. **B.** Cathepsin E. **C.** HIV-1 retropepsin. **D.** Cathepsin B. **E.** Caspase-3. **F.** MMP-2. **G.** MMP-9. **H.** Granzyme B (human). **I.** Cathepsin G. MMP, matrix metallopeptidase. The secondary structure information was extracted from DSSP results. H, helix; E, strand; L, loop.

To better understand the structural determinants surrounding cleavage sites of different proteases, we examined the structural features of protease cleavage sites using the curated PDB structure datasets. Bar graphs for a total of 27 proteases presented in Figure 2 (9 proteases) and Figure S1 (18 proteases) show the secondary structure preferences of protease-specific substrates across the P4–P4′ sites surrounding the cleavage sites. As shown in these figures, different protease cleavage sites generally have distinctly different secondary structure preferences. However, on the other hand, some proteases also share similar secondary structure preferences. For instance, the P4–P4′ site surrounding cleavage sites of caspase-3, granzyme B (human) (Figure 2E and H), cathepsin S, caspase-6, meprin α subunit, meprin β subunit, and LAST_MAM peptidase (Figure S1G, J, and L–N) are more likely to be located in loop regions than in helix and strand regions. In addition, the cleavage sites of most proteases can be found in all three types of secondary structures, except for those of necepsin-1, cathepsin L1 (*Fasciola* sp.), falcipain-2, and falcipain-3 (Figure S1D, F, H, and I). The cleavage sites of these four proteases are predominately found in helix and loop regions, but not in strands. The results are in good agreement with the findings of existing studies and suggest that proteases prefer to cleave within loop regions of substrate proteins, while cleavage within helix/sheet regions is also possible [16], [17], [18]. In addition, we plotted the boxplots for other structural features of positive samples (cleavage sites) for all 27 proteases. These results are provided in supplementary figures, including protrusion index (Figure S2), depth index (Figure S3), solvent accessibility calculated by Naccess (Figures S4−S13), packing (Figure S14), solvent exposure properties (Figures S15 and S16), solvent accessibility calculated by DSSP (Figure S17), backbone torsion angles (Figures S18 and S19), solvent exposure properties (Figures S20–24), B-factor (Figure S25), and hydrogen bonds (Figure S26).

### Performance assessment

**Figure 3:**
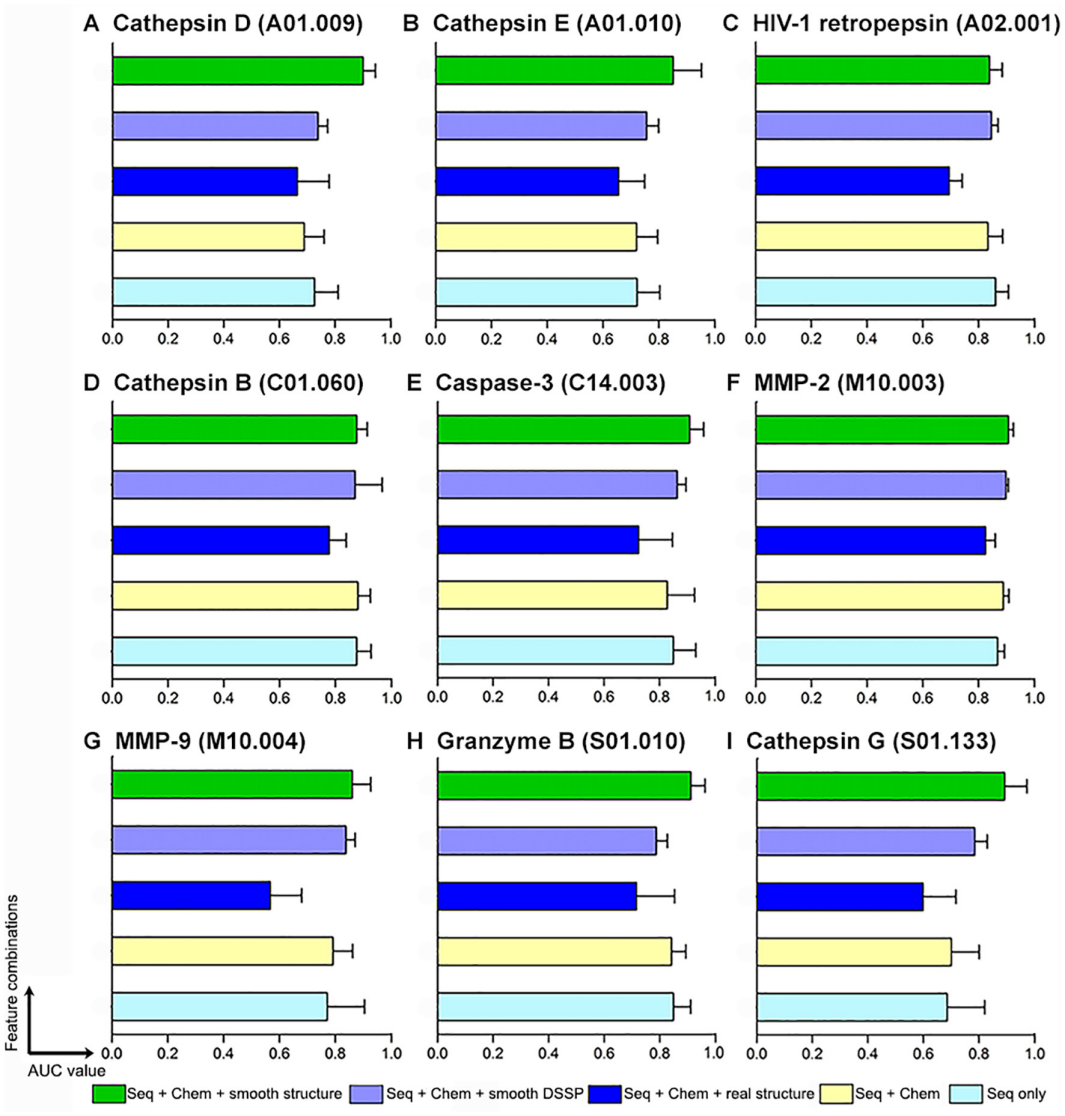
**Performance comparison of CRF models trained using different feature combinations in terms of AUC values** **A.** Cathepsin D. **B.** Cathepsin E. **C.** HIV-1 retropepsin. **D.** Cathepsin B. **E.** Caspase-3. **F.** MMP-2. **G** MMP-9. **H.** Granzyme B (human). **I.** Cathepsin G. The evaluation was based on 10 times of 5-fold cross-validation tests on training datasets.

To examine how the structural features help to predict the cleavage sites and how our proposed feature smoothing algorithm improves the prediction performance of trained CRF models, we evaluated the performance of different types of feature combinations. The experiments were conducted by performing 10 times of 5-fold cross-validation tests using the benchmark datasets. The evaluated features/feature combinations include Seq only (using sequence features only), Seq + Chem (using sequence features together with chemical features), Seq + Chem + real structure (using sequence, chemical, and original structural features, without any smoothing), Seq + Chem + smooth DSSP (using sequence, chemical, and smoothed DSSP structural features), and Seq + Chem + smooth structure (using sequence, chemical, and smoothed structural features). Performance comparisons of different feature combinations in terms of AUC values (average AUC values of 10 times of 5-fold cross-validation tests) are shown in Figure 3 and Table S3.

From these results, several important observations can be made. The Seq + Chem + smooth structure models performed the best compared with all other feature combinations in terms of AUC values for 22 of the 27 tested proteases (see Figure 3 and detailed results in Table S3). Also, the Seq + Chem + smooth DSSP models achieved highest AUC values for meprin β and chymotrypsin A (bovine), while the Seq + Chem models achieved highest AUC values for cathepsin B and lysyl peptidase (bacteria). Seq only model performed the best for HIV-1 retropepsin. These results demonstrate that the sequence features and chemical group features are more relevant and important for the three proteases, while the structural features may not be useful for further improving the cleavage site prediction performance for these proteases. Not surprisingly, the Seq + Chem + real structure models performed the worst among all the compared feature combination models, because the Boolean feature functions of the CRF cannot deal properly with continuous values. This not only leads to the loss of some useful feature information, but also affects the model training.

In addition, to test and verify the statistical significance of AUC improvement by the Seq + Chem + smooth structure models, we conducted a student’s *t*-test to compare the AUC values of different feature combination models trained with CRF. The *P* values of the student’s *t*-test are given in Table 1, indicating that the AUCs of the Seq + Chem + smooth structure models were significantly (*P* ≤0.01, marked in bold) higher than those of other models according to the pairwise tests. Feature combinations that achieved the best performance during each comparison test are underlined in Table 1. Furthermore, the AUC values of the Seq + Chem + smooth DSSP models were significantly higher than those of the Seq + Chem and the Seq + Chem + real structure models, while inconclusive with the Seq only models. Altogether, both the performance comparisons and pairwise *t*-test comparisons demonstrate that structural features smoothed by the LOWESS data smoothing algorithm can greatly help to boost the performance of CRF models. A possible explanation is that the LOWESS smoothing takes the structural variables defined over the cleavage segment P4–P4′ sites, and flattens the fluctuations of the structural variables over the eight AA residues of the cleavage sites. This makes intuitive sense because the structural variables are defined over the crystal structure of the protein, which represents only one of the many conformations that constitute the equilibrium ensemble of the protein in solution. In particular, the cleavage site is generally located on or near the surface of the protein, where the side chains of residues on the surface are particularly prone to fluctuations due to thermal contact with the water [16]. As such, a single value for the structural variables of a given AA residue will not be a fair representation, especially given that in crystal structures, sidechain conformations on the surface are often flush against symmetric repeats of the protein [16]. As such, the smoothing of the structural parameters provides a way to reduce these effects and a more appropriate representation of the structural determinants of cleavage sites.

**Table 1:** ***P* values for pairwise *t*-test comparisons of prediction performance using different feature combinations**

**Feature combination**	***P* value**
Seq + Chem ± smooth DSSP *vs*. Seq only	0.10
Seq + Chem ± smooth DSSP *vs*. Seq + Chem	0.01
Seq + Chem ± smooth DSSP *vs*. Seq + Chem + real structure	1.12E−20
Seq + Chem + smooth structure *vs*. Seq only	1.91E−13
Seq + Chem + smooth structure *vs*. Seq + Chem	2.04E−16
Seq + Chem + smooth structure *vs*. Seq + Chem + real structure	3.94E−49
Seq + Chem + smooth structure *vs*. Seq + Chem + smooth DSSP	5.38E−09

*Note*: Tests were performed using AUC results of 10 times 5-fold cross-validation tests of all the 27 proteases examined.

Moreover, in order to further illustrate the advantage of CRF, we benchmarked the performance of CRF models with that of the other two popular machine learning algorithms, *i.e.*, support vector machine (SVM) and random forest (RF), on both the training and independent test datasets. The performance results on the 5-fold cross validation and independent tests are provided in Tables S3 and S4, respectively. As a result, the CRF models achieved the best performance across almost all comparative experiments on the training datasets. The only exceptions were the Seq + Chem + real structure feature for matrix metallopeptidase 2 (MMP-2) and the Seq + Chem feature for both astacin and meprin α, for which the RF models achieved the best prediction results. For the performance evaluation on the independent test, we applied the SVM and RF models trained using the Seq + Chem + smooth structure feature combinations, as the SVM and RF models trained on this feature combination performed the best compared to all the other feature combinations. The performance results on the independent tests confirm that the CRF models of Procleave achieve overall a better performance than SVM and RF models, for all 27 proteases examined. Taken together, the performance results on both 5-fold cross validation and independent tests demonstrate the superiority of the CRF framework, making it the model of choice for the development of Procleave.

Therefore, we accordingly built two prediction models for protease cleavage site prediction from both protein sequences and structures. We built the Procleave*_*sequence based on Seq + Chem feature combination models for protease cleavage site prediction from protein sequences; while the Procleave*_*smooth based on Seq + Chem + smooth structure feature combination was built for protease cleavage site prediction from protein structures.

### Comparison with existing methods

**Figure 4:**
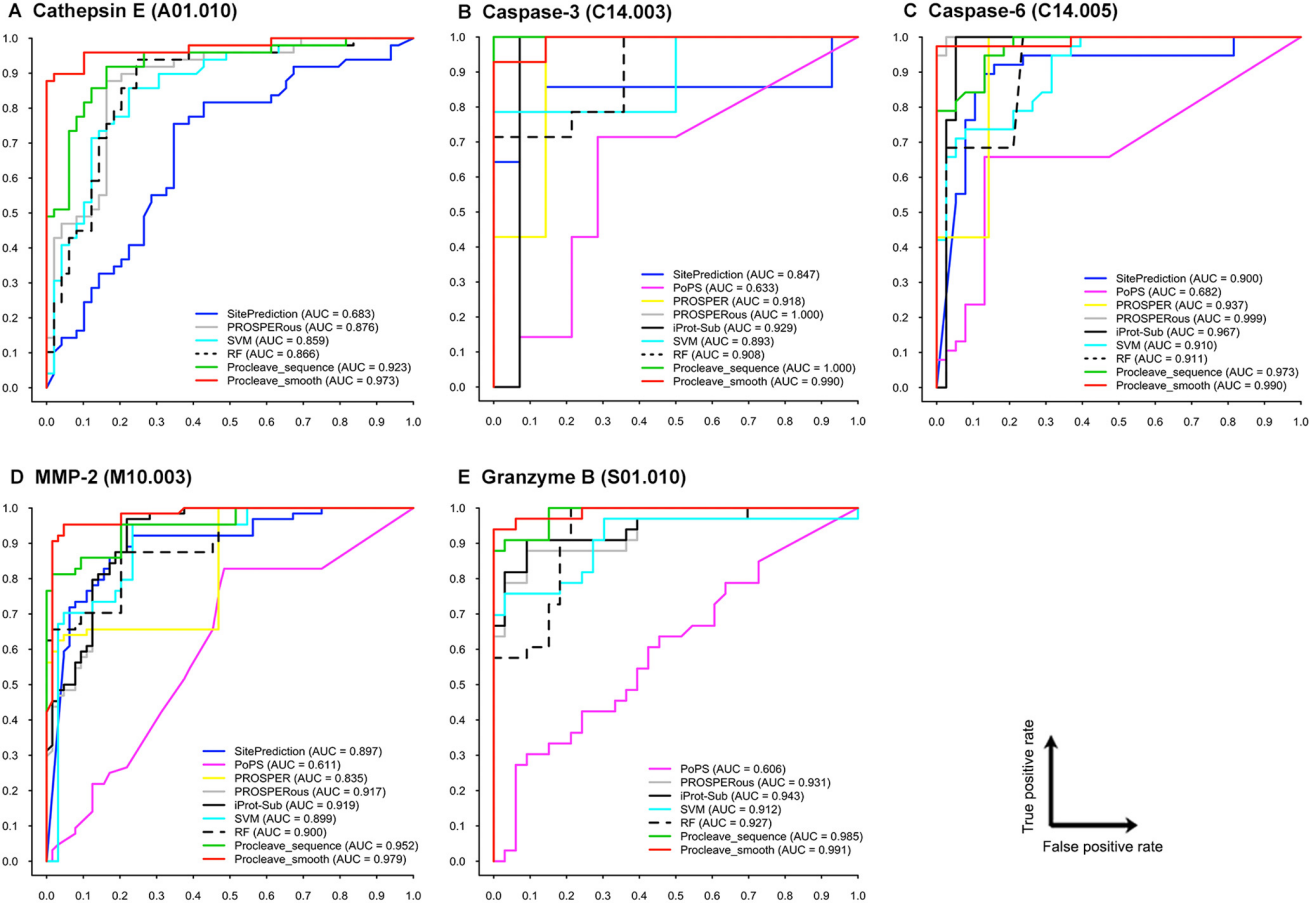
**Comparison of cleavage site prediction performance of Procleave and other methods in terms of AUC values for 5 different proteases** **A.** Cathepsin E. **B.** Caspase-3. **C.** Caspase-6. **D.** MMP-2. **E.** Granzyme B. PoPS, PROSPER, and iProt-Sub cannot predict cleavage sites of cathepsin E; SitePrediction and PROSPER cannot predict cleavage sites of granzyme B. SVM and RF were included to test whether the conditional random field model employed in Procleave provides better performance.

We compared the performance of two variant models ‘Procleave_sequence’ and ‘Procleave_smooth’ against five existing tools, including PoPS, SitePrediction, PROSPER, PROSPERous, and iProt-Sub, by performing the independent test. In order to avoid any potential bias and objectively assess the performance, we submitted the PDB sequences in the FASTA format in the independent test dataset to each of the webservers of these methods. The detailed performance results are summarized in Table S4*.* In addition to AUC, MCC, Acc, Sn, Sp, and precision are also provided and listed in Table S4, while ROC curves are presented in Figure 4.

Figure 4 displays the ROC curves of PoPS, SitePrediction, PROSPER, PROSPERous, iProt-Sub, Procleave_smooth, and Procleave_sequence on the independent test dataset. As the entries in the independent test dataset were obtained solely from the newly identified protease substrates and cleavage sites from the most-recent version of MEROPS (12.0) as compared to its previous version (release 9.0), the amount of newly added data was relatively small, and there was even fewer data remaining after mapping onto the PDB 3D structures. Therefore, only five proteases were used for the test, including cathepsin E, caspase-3, caspase-6, MMP-2, and granzyme B (human). As can be seen, Procleave_smooth (red line) performed the best and Procleave_sequence (green line) ranked second in terms of AUC for Cathepsin E (Figure 4A), MMP-2 (Figure 4D), and granzyme B (human) (Figure 4E). For caspase-3, Procleave_sequence and PROSPERous achieved the best performance (AUC = 1) and Procleave_smooth achieved the second highest AUC (0.990) (Figure 4B). While for caspase-6, PROSPERous achieved the highest AUC (0.999) value and Procleave_smooth ranked second (Figure 4C). To summarize, all these results demonstrate that Procleave is a reliable and powerful bioinformatics approach that improves protease cleavage site prediction. In particular, there are three important factors that account for the good performance of Procleave. First, the high quality and comprehensive 3D structural substrate cleavage data provide solid foundation for the training of Procleave. Second, extracting useful and complementary 3D structural features as calculated by multiple software tools provides a better description of the characteristics of substrate cleavage sites. And lastly, processing initial 3D structural features using the LOWESS data-smoothing algorithm is necessary to enable CRF to learn the underlying rules and characteristics of protease-specific cleavage events.

### Webserver implementation

To facilitate bioinformatics analyses of novel protease target substrates and cleavage sites, we implemented the CRF-based Procleave approach and developed a publicly available webserver for the wider research community. The Procleave webserver was implemented using HTML and Perl. The webserver is freely accessible at http://procleave.erc.monash.edu/. Procleave webserver is operated by Tomcat7 and configured in a Linux server with an eight-core CPU, 500-GB hard disk and 16-GB memory. Both the Procleave_smooth and Procleave_sequence variant models are implemented on the web server. The web server requires two steps of inputs in order to make a prediction of the potential cleavage sites for the given protein. First, Procleave_smooth requires users to supply a protein 3D structure file (*.pdb file is preferred), while for Procleave_sequence models, users are required to input the FASTA formatted protein sequences. Second, users need to specify the PDB chain name and protease type in the case of submitting the 3D structure file. Each submission takes approximately 3–4 min to complete. The prediction outcome for the submitted structure file is returned on the result webpage. The prediction results can be exported in the CSV, Excel, and PDF formats. 3Dmol.js [36] is also employed for protein 3D structure visualization at the webserver. The predicted potential cleavage sites are labelled at their corresponding positions.

### Structural proteome-wide prediction

Furthermore, we conducted a structural proteome-wide prediction of novel protease substrate cleavage sites (containing 17,628 human proteins extracted from the PDB database) by applying the Procleave_smooth model. The results are briefly summarized in this section. We applied an Sp threshold of 99% to all predictions [15], [31], [37], [38] to generate a compendium of high-confidence predicted cleavage sites and then performed the statistical analyses. Statistics of the identified cleavage substrates and the predicted cleavage sites for the 27 different proteases are summarized in Table S5. The results of the identified cleavage substrates and their cleavage sites are also accessible at the Procleave webserver, which can be freely downloaded at http://procleave.erc.monash.edu/.

### Case study

**Figure 5:**
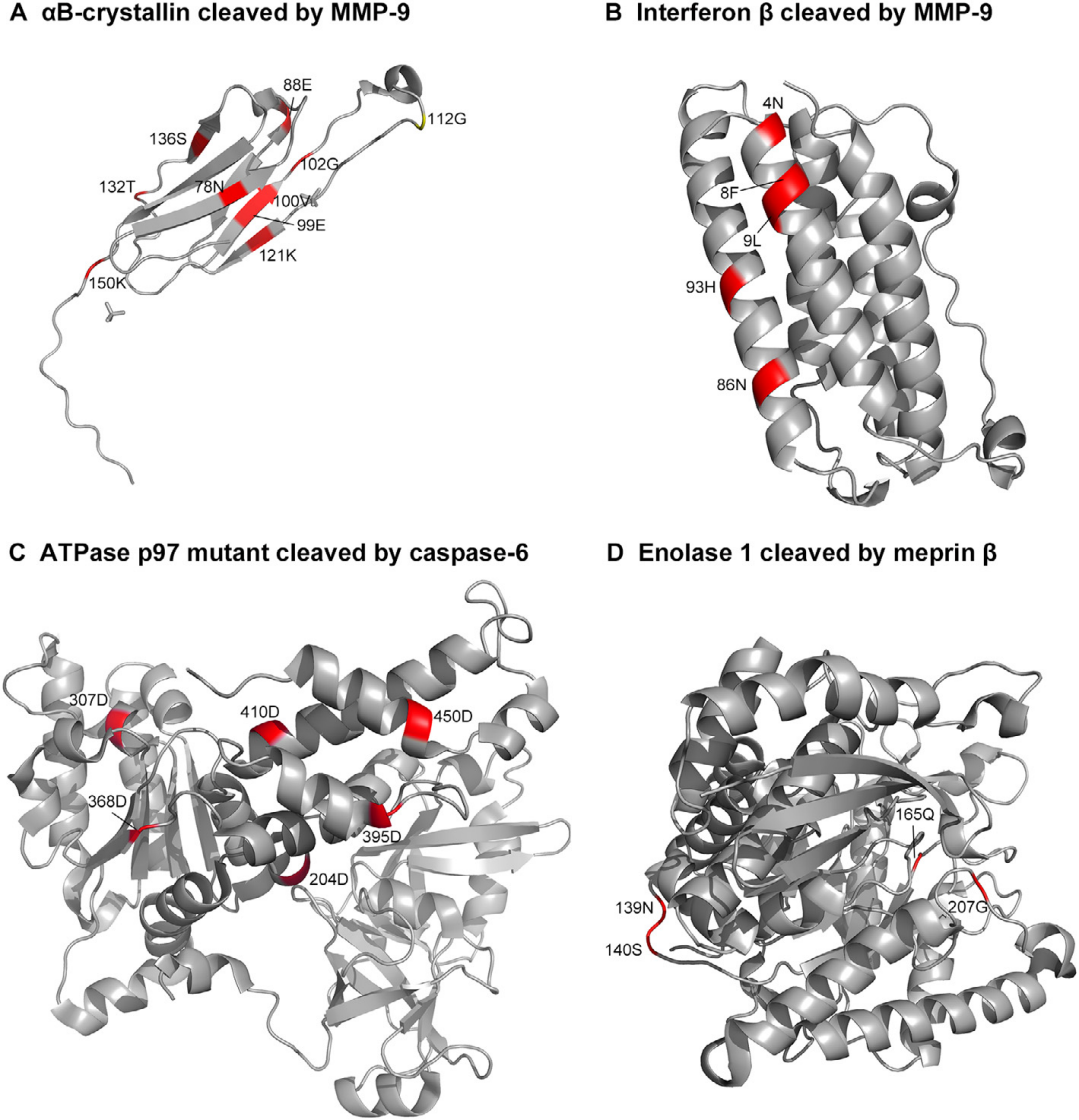
**Predicted cleavage sites of four substrate protein structures** **A.** Human αB crystalline (PDB ID: 3L1G, chain: A) cleaved by MMP-9. **B.** Human Interferon β (PDB ID: 1AU1, chain: A) cleaved by MMP-9. **C.** ATPase p97 mutant (PDB ID: 3HU2, chain A) cleaved by caspase-6. **D.** Human enolase 1 (PDB ID: 3B97, chain A) cleaved by meprin β.

To illustrate the utility and capacity of Procleave, a case study of the protease-specific cleavage site prediction in four substrate proteins was conducted in this section. The four proteins were selected from the independent test dataset. The first protein is human αB crystalline (PDB ID: 3L1G, chain A), which functions as a chaperone and oligomeric assembly. It serves as a stability sensor and can recognize and bind to destabilized proteins in eye lens and other tissues [39]. The second protein is human interferon β (PDB ID:1AU1, chain A), which is the protein to defend the cells from various viruses [40]. The third protein is an ATPase p97 mutant (PDB ID: 3HU2, chain A). ATPase p97 is one of the most abundant cytosolic proteins and can interact with different adaptor proteins involved in many cellular activities, including protein degradation, cell cycle regulation, and membrane fusion [41]. The fourth protein is human enolase 1 (PDB ID: 3B97, chain A), which is a glycolytic enzyme expressed in most tissues. A previous study indicates that this protein is involved in many diseases, including metastatic cancer, ischaemia, autoimmune disorders, and bacterial infection [42]. Structure scanning results and the predicted cleavage sites are shown in Figure 5 and Table S6. All correctly predicted cleavage sites are highlighted in red. These prediction results of demonstrate that Procleave could correctly identify all the experimentally verified cleavage sites. These results suggest that Procleave is a useful tool and can be used to identify cleavage sites based on the 3D structural information of the substrate proteins.

## Conclusion

In the present work, we developed Procleave, a new CRF approach, which combines both sequence and structural information to enhance the protease-specific cleavage site prediction. Procleave employs multi-faceted 3D structure-based features, in combination with a LOWESS smoothing optimization algorithm to train and optimize the CRF-based cleavage site prediction models for a protease. We conducted a comprehensive set of empirical benchmarking tests to benchmark the performance of CRF models built based on different combinations of sequence, chemical, and structural features. We also assessed the performance of Procleave with several state-of-the-art approaches. The comparison results demonstrate that Procleave outperforms these methods, and the LOWESS smoothing optimization is critical to the performance of Procleave. The aim of this study is to systematically investigate whether both sequence-derived and real 3D structural information can be integrated in a machine learning framework to improve the substrate cleavage site prediction for 27 major proteases. A user-friendly webserver of Procleave has been made available as an implementation of the proposed approach. All predicted cleavage sites of the human proteome with 3D the structure data available are provided for further protease biology research. We envisage that Procleave will become a useful tool in the future, facilitating community-wide hypothesis-driven experimental design and functional characterization studies. As a generally useful framework, the CRF-based methodology combined with the LOWESS smoothing optimization algorithm can be readily extended and applied to develop useful methods for predicting other important types of PTM sites [43], [44], [45], [46] and functional sites that utilize 3D structural information in future work.

## Authors’ contributions

JS, JL, and TML conceived the project and supervised the study. JS, FL, and AL designed the algorithm and drafted the manuscript. FL performed the machine learning experiments and analyzed the results. FL and YW analyzed the performance comparison results. FL, QL, and DX implemented the online webserver. TA, GIW, and AIS revised the manuscript critically for important intellectual content. All authors read, revised, and approved the final manuscript.

## Competing interests

The authors have declared no competing interests.

## Data Availability

The datasets and proteome-wide prediction results are publicly accessible at http://procleave.erc.monash.edu/gallery.html/.

## Supplementary material

Supplementary Table S6

Supplementary Figure S1 **Structural determinants of the substrate specificity of 18 proteases across the P4–P4′ cleavage sites. A.** Pepsin A. **B.** Rhizopuspepsin. **C.** Aspergillopepsin. **D.** Necepsin-1. **E.** Cathepsin L. **F.** Cathepsin L1 (*Fasciola* sp.). **G.** Cathepsin S. **H.** Falcipain-2. **I.** Falcipain-3. **J.** Caspase-6. **K.** Astacin. **L.** Meprin alpha subunit. **M.** Meprin beta subunit. **N.** LAST_MAM peptidase. **O.** Chymotrypsin A. **P.** Elastase-2. **Q.** Glutamyl peptidase I. **R.** Lysyl peptidase (bacteria). H, helix; E, strand; L, loop.

Supplementary Figure S2 Boxplots of protrusion index calculated by CX.

Supplementary Figure S3 Boxplots of depth index calculated by DPX.

Supplementary Figure S4 Boxplots of all-atom absolute solvent accessibility calculated by Naccess.

Supplementary Figure S5 Boxplots of all-atom relative solvent accessibility calculated by Naccess.

Supplementary Figure S6 Boxplots of total-side absolute solvent accessibility calculated by Naccess.

Supplementary Figure S7 Boxplots of total-side relative solvent accessibility calculated by Naccess.

Supplementary Figure S8 Boxplots of main-chain absolute solvent accessibility calculated by Naccess.

Supplementary Figure S9 Boxplots of main-chain relative solvent accessibility calculated by Naccess.

Supplementary Figure S10 Boxplots of non-polar absolute solvent accessibility calculated by Naccess.

Supplementary Figure S11 Boxplots of non-polar relative solvent accessibility calculated by Naccess.

Supplementary Figure S12 Boxplots of all polar absolute solvent accessibility calculated by Naccess.

Supplementary Figure S13 Boxplots of all polar relative solvent accessibility calculated by Naccess.

Supplementary Figure S14 Boxplots of packing feature.

Supplementary Figure S15 Boxplots of molecular solvent-excluded surfaces calculated by MSMS.

Supplementary Figure S16 Boxplots of molecular solvent-accessible surfaces calculated by MSMS.

Supplementary Figure S17 Boxplots of solvent accessibility calculated by DSSP.

Supplementary Figure S18 Boxplots of torsion angle (PHI) calculated by DSSP.

Supplementary Figure S19 Boxplots of torsion angle (PSI) calculated by DSSP.

Supplementary Figure S20 Boxplots of contact number calculated by Biopython.

Supplementary Figure S21 Boxplots of HSEAU calculated by Biopython.

Supplementary Figure S22 Boxplots of HSEAD calculated by Biopython.

Supplementary Figure S23 Boxplots of HSEBU calculated by Biopython.

Supplementary Figure S24 Boxplots of HSEBD calculated by Biopython.

Supplementary Figure S25 Boxplots of B-factor extracted from PDB files.

Supplementary Figure S26 Boxplots of hydrogen bond calculated by HBPLUS.
