# Supplementary material for: Procleave: Predicting Protease-specific Substrate Cleavage Sites by Combining Sequence and Structural Information
Source: Genomics Proteomics Bioinformatics. 2020 May 12;18(1):52–64. doi: 10.1016/j.gpb.2019.08.002 (PMC7393547; doi:10.1016/j.gpb.2019.08.002)
Supplement: Supplementary Table S1 [file mmc1.docx]

**Table S1**  **Statistics of the curated benchmark substrate structure dataset for the 27 proteases examined in this study**

| **Family** | **Protease** | **MEROPS ID** | **No. of substrate structures** | **No. of cleavage sites** |
| --- | --- | --- | --- | --- |
| Aspartic | Pepsin A | A01.001 | 15 | 292 |
|  | Cathepsin D | A01.009 | 43 | 122 |
|  | Cathepsin E | A01.010 | 44 | 113 |
|  | Rhizopuspepsin | A01.012 | 5 | 192 |
|  | Aspergillopepsin I | A01.016 | 7 | 128 |
|  | Necepsin-1 | A01.053 | 3 | 108 |
|  | HIV-1 retropepsin | A02.001 | 91 | 125 |
| Cysteine | Cathepsin L | C01.032 | 105 | 157 |
|  | Cathepsin L1 (*Fasciola* sp.) | C01.033 | 4 | 168 |
|  | Cathepsin S | C01.034 | 90 | 124 |
|  | Falcipain-2 | C01.046 | 2 | 118 |
|  | Cathepsin B | C01.060 | 82 | 139 |
|  | Falcipain-3 | C01.063 | 2 | 96 |
|  | Caspase-3 | C14.003 | 46 | 51 |
|  | Caspase-6 | C14.005 | 16 | 39 |
| Metallo | MMP-2 | M10.003 | 209 | 458 |
|  | MMP-9 | M10.004 | 22 | 62 |
|  | Astacin | M12.001 | 52 | 62 |
|  | Meprin alpha | M12.002 | 52 | 92 |
|  | Meprin beta | M12.004 | 71 | 94 |
|  | LAST_MAM peptidase | M12.033 | 83 | 136 |
| Serine | Chymotrypsin A (bovine) | S01.001 | 78 | 258 |
|  | Granzyme B (human) | S01.010 | 58 | 74 |
|  | Elastase-2 | S01.131 | 66 | 117 |
|  | Cathepsin G | S01.133 | 50 | 71 |
|  | Glutamyl peptidase-1 | S01.269 | 161 | 303 |
|  | Lysyl peptidase (bacteria) | S01.280 | 41 | 60 |

*Note*: CD-HIT program was used to cluster the sequences to reduce the sequence identity to 70% for this structure dataset. MMP-2, matrix metallopeptidase 2; MMP-9, matrix metallopeptidase 9.
