# Supplementary material for: Procleave: Predicting Protease-specific Substrate Cleavage Sites by Combining Sequence and Structural Information
Source: Genomics Proteomics Bioinformatics. 2020 May 12;18(1):52–64. doi: 10.1016/j.gpb.2019.08.002 (PMC7393547; doi:10.1016/j.gpb.2019.08.002)
Supplement: Supplementary Table S2 [file mmc2.docx]

**Table S2 Statistics of the independent test curated substrate structure dataset**

| **Protease** | **MEROPS ID** | **No. of substrate structures** | **No. of cleavage sites** |
| --- | --- | --- | --- |
| Cathepsin E | A01.010 | 16 | 49 |
| Caspase-3 | C14.003 | 11 | 14 |
| Caspase-6 | C14.005 | 26 | 38 |
| MMP-2 | M10.003 | 23 | 64 |
| Granzyme B (human) | S01.010 | 28 | 33 |
