# Supplementary material for: Procleave: Predicting Protease-specific Substrate Cleavage Sites by Combining Sequence and Structural Information
Source: Genomics Proteomics Bioinformatics. 2020 May 12;18(1):52–64. doi: 10.1016/j.gpb.2019.08.002 (PMC7393547; doi:10.1016/j.gpb.2019.08.002)
Supplement: Supplementary Table S3 [file mmc3.docx]

**Table S3**  **AUC values of substrate cleavage site prediction for the 27 proteases using 5 different sequence encoding schemes**

| **Protease** | **Method** | **Seq only** | **Seq + Chem** | **Seq + Chem + real structure** | **Seq+ Chem + smooth DSSP** | **Seq + Chem + smooth structure** |
| --- | --- | --- | --- | --- | --- | --- |
| Pepsin A | Procleave | 0.590 ± 0.04 | 0.570 ± 0.03 | 0.579 ± 0.04 | 0.612 ± 0.02 | **0.694 ± 0.04** |
|  | SVM | 0.553 ± 0.07 | 0.542 ± 0.04 | 0.548 ± 0.06 | 0.595 ± 0.07 | 0.624 ± 0.05 |
|  | RF | 0.572 ± 0.05 | 0.562 ± 0.06 | 0.569 ± 0.11 | 0.599 ± 0.03 | 0.643 ± 0.11 |
| Cathepsin D | Procleave | 0.727 ± 0.08 | 0.690 ± 0.07 | 0.664 ± 0.11 | 0.739 ± 0.03 | **0.900 ± 0.04** |
|  | SVM | 0.692 ± 0.13 | 0.639 ± 0.12 | 0.660 ± 0.10 | 0.732 ± 0.01 | 0.872 ± 0.07 |
|  | RF | 0.707 ± 0.09 | 0.657 ± 0.08 | 0.657 ± 0.09 | 0.726 ± 0.02 | 0.852 ± 0.04 |
| Cathepsin E | Procleave | 0.722 ± 0.08 | 0.720 ± 0.07 | 0.655 ± 0.09 | 0.756 ± 0.04 | **0.851 ± 0.10** |
|  | SVM | 0.706 ± 0.03 | 0.702 ± 0.09 | 0.627 ± 0.13 | 0.725 ± 0.07 | 0.813 ± 0.09 |
|  | RF | 0.711 ± 0.09 | 0.710 ± 0.12 | 0.635 ± 0.06 | 0.744 ± 0.03 | 0.835 ± 0.13 |
| Rhizopuspepsin | Procleave | 0.509 ± 0.05 | 0.508 ± 0.04 | 0.540 ± 0.04 | 0.568 ± 0.03 | **0.655 ± 0.03** |
|  | SVM | 0.505 ± 0.04 | 0.504 ± 0.06 | 0.531 ± 0.03 | 0.548 ± 0.03 | 0.626 ± 0.08 |
|  | RF | 0.501 ± 0.09 | 0.503 ± 0.07 | 0.539 ± 0.04 | 0.561 ± 0.02 | 0.634 ± 0.04 |
| Aspergillopepsin I | Procleave | 0.668 ± 0.08 | 0.656 ± 0.08 | 0.682 ± 0.09 | 0.736 ± 0.04 | **0.833 ± 0.09** |
|  | SVM | 0.649 ± 0.08 | 0.648 ± 0.09 | 0.674 ± 0.08 | 0.728 ± 0.06 | 0.820 ± 0.07 |
|  | RF | 0.664 ± 0.06 | 0.656 ± 0.11 | 0.676 ± 0.09 | 0.731 ± 0.04 | 0.826 ± 0.11 |
| Necepsin-1 | Procleave | 0.775 ± 0.09 | 0.756 ± 0.10 | 0.783 ± 0.08 | 0.870 ± 0.03 | **0.965 ± 0.03** |
|  | SVM | 0.745 ± 0.10 | 0.737 ± 0.13 | 0.747 ± 0.07 | 0.835 ± 0.02 | 0.942 ± 0.02 |
|  | RF | 0.765 ± 0.09 | 0.742 ± 0.08 | 0.755 ± 0.10 | 0.829 ± 0.05 | 0.957 ± 0.03 |
| HIV-1 retropepsin | Procleave | **0.860 ± 0.05** | 0.833 ± 0.05 | 0.694 ± 0.04 | 0.846 ± 0.02 | 0.839 ± 0.04 |
|  | SVM | 0.838 ± 0.06 | 0.819 ± 0.07 | 0.675 ± 0.11 | 0.817 ± 0.04 | 0.819 ± 0.07 |
|  | RF | 0.849 ± 0.03 | 0.828 ± 0.05 | 0.679 ± 0.06 | 0.824 ± 0.03 | 0.826 ± 0.05 |
| Cathepsin L | Procleave | 0.845 ± 0.03 | 0.820 ± 0.03 | 0.735 ± 0.08 | 0.821 ± 0.02 | **0.853 ± 0.05** |
|  | SVM | 0.818 ± 0.01 | 0.808 ± 0.02 | 0.721 ± 0.11 | 0.810 ± 0.03 | 0.825 ± 0.07 |
|  | RF | 0.826 ± 0.06 | 0.815 ± 0.02 | 0.729 ± 0.09 | 0.814 ± 0.02 | 0.833 ± 0.03 |
| Cathepsin L1 (*Fasciola* sp.) | Procleave | 0.938 ± 0.06 | 0.930 ± 0.06 | 0.933 ± 0.06 | 0.981 ± 0.01 | **0.994 ± 0.01** |
|  | SVM | 0.899 ± 0.09 | 0.885 ± 0.04 | 0.900 ± 0.04 | 0.913 ± 0.02 | 0.925 ± 0.01 |
|  | RF | 0.907 ± 0.07 | 0.901 ± 0.09 | 0.905 ± 0.11 | 0.922 ± 0.06 | 0.934 ± 0.03 |
| Cathepsin S | Procleave | 0.870 ± 0.05 | 0.866 ± 0.06 | 0.783 ± 0.04 | 0.845 ± 0.03 | **0.901 ± 0.03** |
|  | SVM | 0.840 ± 0.04 | 0.822 ± 0.04 | 0.769 ± 0.02 | 0.823 ± 0.09 | 0.857 ± 0.01 |
|  | RF | 0.859 ± 0.02 | 0.853 ± 0.05 | 0.764 ± 0.07 | 0.827 ± 0.06 | 0.886 ± 0.04 |
| Falcipain-2 | Procleave | 0.964 ± 0.03 | 0.959 ± 0.04 | 0.953 ± 0.05 | 0.970 ± 0.01 | **0.987 ± 0.02** |
|  | SVM | 0.922 ± 0.02 | 0.920 ± 0.04 | 0.917 ± 0.08 | 0.933 ± 0.02 | 0.951 ± 0.11 |
|  | RF | 0.938 ± 0.07 | 0.931 ± 0.01 | 0.917 ± 0.03 | 0.940 ± 0.07 | 0.955 ± 0.04 |
| Cathepsin B | Procleave | 0.876 ± 0.05 | **0.880 ± 0.04** | 0.777 ± 0.06 | 0.870 ± 0.09 | 0.876 ± 0.04 |
|  | SVM | 0.848 ± 0.01 | 0.851 ± 0.09 | 0.725 ± 0.01 | 0.819 ± 0.06 | 0.826 ± 0.07 |
|  | RF | 0.854 ± 0.02 | 0.856 ± 0.05 | 0.746 ± 0.07 | 0.837 ± 0.01 | 0.841 ± 0.06 |
| Falcipain-3 | Procleave | 0.934 ± 0.06 | 0.899 ± 0.08 | 0.876 ± 0.10 | 0.941 ± 0.02 | **0.988 ± 0.02** |
|  | SVM | 0.905 ± 0.11 | 0.848 ± 0.01 | 0.829 ± 0.07 | 0.912 ± 0.01 | 0.928 ± 0.06 |
|  | RF | 0.913 ± 0.05 | 0.863 ± 0.08 | 0.841 ± 0.02 | 0.924 ± 0.06 | 0.935 ± 0.03 |
| Caspase-3 | Procleave | 0.850 ± 0.08 | 0.828 ± 0.10 | 0.725 ± 0.12 | 0.864 ± 0.03 | **0.909 ± 0.05** |
|  | SVM | 0.823 ± 0.05 | 0.808 ± 0.11 | 0.687 ± 0.15 | 0.853 ± 0.01 | 0.899 ± 0.03 |
|  | RF | 0.828 ± 0.07 | 0.823 ± 0.14 | 0.717 ± 0.09 | 0.864 ± 0.06 | 0.904 ± 0.08 |
| Caspase-6 | Procleave | 0.890 ± 0.14 | 0.858 ± 0.18 | 0.684 ± 0.16 | 0.852 ± 0.05 | **0.930 ± 0.06** |
|  | SVM | 0.839 ± 0.17 | 0.813 ± 0.16 | 0.661 ± 0.22 | 0.839 ± 0.07 | 0.902 ± 0.07 |
|  | RF | 0.857 ± 0.13 | 0.830 ± 0.11 | 0.625 ± 0.19 | 0.848 ± 0.09 | 0.893 ± 0.03 |
| MMP-2 | Procleave | 0.868 ± 0.02 | 0.888 ± 0.02 | 0.824 ± 0.04 | 0.898 ± 0.01 | **0.907 ± 0.02** |
|  | SVM | 0.850 ± 0.03 | 0.864 ± 0.01 | 0.813 ± 0.02 | 0.858 ± 0.06 | 0.884 ± 0.01 |
|  | RF | 0.842 ± 0.02 | 0.860 ± 0.04 | **0.825 ± 0.01** | 0.876 ± 0.04 | 0.891 ± 0.03 |
| MMP-9 | Procleave | 0.771 ± 0.13 | 0.791 ± 0.07 | 0.567 ± 0.11 | 0.838 ± 0.03 | **0.860 ± 0.06** |
|  | SVM | 0.736 ± 0.16 | 0.760 ± 0.03 | 0.554 ± 0.08 | 0.826 ± 0.01 | 0.851 ± 0.07 |
|  | RF | 0.752 ± 0.11 | 0.777 ± 0.09 | 0.562 ± 0.12 | 0.830 ± 0.01 | 0.855 ± 0.06 |
| Astacin | Procleave | 0.811 ± 0.08 | 0.778 ± 0.09 | 0.645 ± 0.11 | 0.775 ± 0.05 | **0.888 ± 0.09** |
|  | SVM | 0.798 ± 0.07 | 0.778 ± 0.11 | 0.644 ± 0.08 | 0.753 ± 0.06 | 0.839 ± 0.10 |
|  | RF | 0.808 ± 0.04 | **0.782 ± 0.13** | 0.638 ± 0.10 | 0.772 ± 0.09 | 0.852 ± 0.13 |
| Meprin alpha | Procleave | 0.757 ± 0.09 | 0.705 ± 0.10 | 0.693 ± 0.10 | 0.750 ± 0.02 | **0.873 ± 0.07** |
|  | SVM | 0.718 ± 0.10 | 0.678 ± 0.09 | 0.656 ± 0.03 | 0.731 ± 0.01 | 0.829 ± 0.10 |
|  | RF | 0.742 ± 0.15 | **0.707 ± 0.07** | 0.691 ± 0.11 | 0.746 ± 0.04 | 0.864 ± 0.13 |
| Meprin beta | Procleave | 0.817 ± 0.06 | 0.802 ± 0.05 | 0.663 ± 0.07 | **0.825 ± 0.08** | 0.804 ± 0.09 |
|  | SVM | 0.793 ± 0.03 | 0.779 ± 0.02 | 0.621 ± 0.09 | 0.793 ± 0.07 | 0.782 ± 0.11 |
|  | RF | 0.802 ± 0.02 | 0.799 ± 0.06 | 0.635 ± 0.04 | 0.809 ± 0.11 | 0.799 ± 0.08 |
| LAST_MAM peptidase | Procleave | 0.826 ± 0.05 | 0.814 ± 0.05 | 0.674 ± 0.07 | 0.792 ± 0.04 | **0.846 ± 0.06** |
|  | SVM | 0.802 ± 0.02 | 0.791 ± 0.04 | 0.654 ± 0.08 | 0.779 ± 0.03 | 0.825 ± 0.05 |
|  | RF | 0.806 ± 0.04 | 0.799 ± 0.11 | 0.660 ± 0.02 | 0.782 ± 0.07 | 0.843 ± 0.09 |
| Chymotrypsin A (bovine) | Procleave | 0.670 ± 0.06 | 0.686 ± 0.06 | 0.679 ± 0.04 | **0.782 ± 0.03** | 0.723 ± 0.04 |
|  | SVM | 0.656 ± 0.03 | 0.675 ± 0.04 | 0.666 ± 0.09 | 0.769 ± 0.01 | 0.712 ± 0.01 |
|  | RF | 0.665 ± 0.05 | 0.684 ± 0.08 | 0.675 ± 0.04 | 0.774 ± 0.06 | 0.713 ± 0.03 |
| Granzyme B (human) | Procleave | 0.849 ± 0.06 | 0.842 ± 0.05 | 0.716 ± 0.14 | 0.788 ± 0.04 | **0.912 ± 0.05** |
|  | SVM | 0.833 ± 0.07 | 0.826 ± 0.02 | 0.703 ± 0.17 | 0.761 ± 0.03 | 0.889 ± 0.03 |
|  | RF | 0.830 ± 0.09 | 0.823 ± 0.06 | 0.708 ± 0.11 | 0.769 ± 0.11 | 0.894 ± 0.08 |
| Elastase-2 | Procleave | 0.752 ± 0.09 | 0.757 ± 0.06 | 0.609 ± 0.09 | 0.778 ± 0.03 | **0.818 ± 0.07** |
|  | SVM | 0.730 ± 0.11 | 0.735 ± 0.03 | 0.602 ± 0.13 | 0.759 ± 0.07 | 0.790 ± 0.03 |
|  | RF | 0.732 ± 0.09 | 0.737 ± 0.05 | 0.608 ± 0.06 | 0.762 ± 0.01 | 0.800 ± 0.01 |
| Cathepsin G | Procleave | 0.685 ± 0.13 | 0.700 ± 0.10 | 0.598 ± 0.12 | 0.784 ± 0.04 | **0.891 ± 0.08** |
|  | SVM | 0.615 ± 0.16 | 0.676 ± 0.13 | 0.532 ± 0.11 | 0.766 ± 0.02 | 0.861 ± 0.04 |
|  | RF | 0.619 ± 0.11 | 0.683 ± 0.08 | 0.554 ± 0.07 | 0.779 ± 0.01 | 0.889 ± 0.06 |
| Glutamyl peptidase I | Procleave | 0.612 ± 0.08 | 0.612 ± 0.08 | 0.606 ± 0.04 | 0.707 ± 0.02 | **0.760 ± 0.03** |
|  | SVM | 0.592 ± 0.06 | 0.594 ± 0.09 | 0.597 ± 0.06 | 0.681 ± 0.04 | 0.723 ± 0.07 |
|  | RF | 0.608 ± 0.03 | 0.606 ± 0.07 | 0.602 ± 0.01 | 0.687 ± 0.01 | 0.738 ± 0.02 |
| Lysyl peptidase (bacteria) | Procleave | 0.603 ± 0.07 | **0.626 ± 0.10** | 0.561 ± 0.05 | 0.529 ± 0.06 | 0.612 ± 0.08 |
|  | SVM | 0.587 ± 0.06 | 0.602 ± 0.14 | 0.534 ± 0.02 | 0.515 ± 0.05 | 0.602 ± 0.13 |
|  | RF | 0.594 ± 0.04 | 0.613 ± 0.11 | 0.542 ± 0.06 | 0.527 ± 0.04 | 0.610 ± 0.07 |

*Note*: The results were expressed as mean ± standard deviation (highest AUC values for the respective proteases are marked in bold). MMP, matrix metallopeptidase; SVM, support vector machine; RF, random forest.
