# Supplementary material for: Procleave: Predicting Protease-specific Substrate Cleavage Sites by Combining Sequence and Structural Information
Source: Genomics Proteomics Bioinformatics. 2020 May 12;18(1):52–64. doi: 10.1016/j.gpb.2019.08.002 (PMC7393547; doi:10.1016/j.gpb.2019.08.002)
Supplement: Supplementary Table S4 [file mmc4.docx]

**Table S4 Performance comparison of Procleave_smooth, Procleave_sequence, and 5 other methods for predicting substrate cleavage sites of 5 different proteases**

| **Protease** | **Tool** | **AUC** | **MCC** | **Acc** | **Sn** | **Sp** | **Precision** |
| --- | --- | --- | --- | --- | --- | --- | --- |
| Cathepsin E (A01.010) | SitePrediction | 0.683 | 0.410 | 0.704 | 0.755 | 0.653 | 0.685 |
|  | PROSPERous | 0.876 | 0.410 | 0.694 | 0.531 | 0.857 | 0.788 |
|  | SVM | 0.859 | 0.635 | 0.816 | 0.857 | 0.776 | 0.792 |
|  | RF | 0.866 | 0.654 | 0.827 | 0.857 | 0.796 | 0.808 |
|  | Procleave_sequence | 0.923 | 0.758 | 0.878 | 0.918 | 0.837 | 0.849 |
|  | Procleave_smooth | **0.973** | **0.880** | **0.939** | **0.898** | **0.98** | **0.978** |
| Caspase-3  (C14.003) | SitePrediction | 0.847 | 0.714 | 0.857 | 0.857 | 0.857 | 0.857 |
|  | PoPS | 0.633 | 0.429 | 0.714 | 0.714 | 0.714 | 0.714 |
|  | PROSPER | 0.918 | 0.866 | 0.929 | 1.000 | 0.857 | 0.875 |
|  | PROSPERous | 1.000 | 0.931 | 0.964 | 1.000 | 0.929 | 0.933 |
|  | iProt-Sub | 0.928 | 0.931 | 0.964 | 1.000 | 0.929 | 0.933 |
|  | SVM | 0.893 | 0.804 | 0.893 | 0.786 | 1.000 | 1.000 |
|  | RF | 0.908 | 0.745 | 0.857 | 0.714 | 1.000 | 1.000 |
|  | Procleave_sequence | **1.000** | **1.000** | **1.000** | **1.000** | **1.000** | **1.000** |
|  | Procleave_smooth | 0.990 | 0.931 | 0.964 | 0.929 | 1.000 | 1.000 |
| Caspase-6  (C14.005) | SitePrediction | 0.900 | 0.763 | 0.882 | 0.895 | 0.868 | 0.872 |
|  | PoPS | 0.682 | 0.538 | 0.763 | 0.658 | 0.868 | 0.833 |
|  | PROSPER | 0.937 | 0.830 | 0.908 | 1.000 | 0.816 | 0.844 |
|  | PROSPERous | 0.999 | 0.974 | 0.987 | 1.000 | 0.974 | 0.974 |
|  | iProt-Sub | 0.967 | 0.949 | 0.974 | 1.000 | 0.947 | 0.950 |
|  | SVM | 0.910 | 0.669 | 0.829 | 0.737 | 0.921 | 0.903 |
|  | RF | 0.911 | 0.786 | 0.882 | 1.000 | 0.763 | 0.809 |
|  | Procleave_sequence | 0.973 | 0.791 | 0.895 | 0.921 | 0.868 | 0.875 |
|  | Procleave_smooth | **0.990** | **0.974** | **0.987** | **0.974** | **1.000** | **1.000** |
| MMP-2 (M10.003) | SitePrediction | 0.897 | 0.676 | 0.836 | 0.891 | 0.781 | 0.803 |
|  | PoPS | 0.611 | 0.362 | 0.672 | 0.828 | 0.516 | 0.631 |
|  | PROSPER | 0.835 | 0.563 | 0.773 | 0.656 | 0.891 | 0.857 |
|  | PROSPERous | 0.917 | 0.728 | 0.859 | 0.938 | 0.781 | 0.811 |
|  | iProt-Sub | 0.919 | 0.728 | 0.859 | 0.938 | 0.781 | 0.811 |
|  | SVM | 0.899 | 0.732 | 0.859 | 0.953 | 0.766 | 0.803 |
|  | RF | 0.900 | 0.674 | 0.836 | 0.875 | 0.797 | 0.812 |
|  | Procleave_sequence | 0.952 | 0.910 | 0.953 | 1.000 | 0.906 | 0.914 |
|  | Procleave_smooth | **0.979** | **0.906** | **0.953** | **0.953** | **0.953** | **0.953** |
| Granzyme B (human) (S01.010) | PoPS | 0.606 | 0.182 | 0.591 | 0.606 | 0.576 | 0.588 |
|  | PROSPERous | 0.931 | 0.788 | 0.894 | 0.879 | 0.909 | 0.906 |
|  | iProt-Sub | 0.943 | 0.818 | 0.909 | 0.909 | 0.909 | 0.909 |
|  | SVM | 0.912 | 0.744 | 0.864 | 0.758 | 0.970 | 0.962 |
|  | RF | 0.927 | 0.730 | 0.864 | 0.909 | 0.818 | 0.833 |
|  | Procleave_sequence | 0.985 | 0.880 | 0.939 | 0.909 | 0.970 | 0.968 |
|  | Procleave_smooth | **0.991** | **0.941** | **0.970** | **0.940** | **1.000** | **1.000** |

*Note*: The prediction performance of different methods on cathepsin E, caspase-3, caspase-6, MMP-2, and granzyme B (human) was evaluated on the independent test datasets. The prediction methods tested include SitePrediction, PoPS, PROPSER, PROSPERous, and iProt-Sub. PoPS, PROSPER, and iProt-Sub cannot predict cleavage sites of cathepsin E; SitePrediction and PROSPER cannot predict cleavage sites of granzyme B. SVM and RF were included to test whether the conditional random field model employed in Procleave provides better performance. AUC, area under the curve; MCC, Matthew’s correlation coefficient; Acc, accuracy; Sn, sensitivity; Sp, specificity.
