# Supplementary material for: Procleave: Predicting Protease-specific Substrate Cleavage Sites by Combining Sequence and Structural Information
Source: Genomics Proteomics Bioinformatics. 2020 May 12;18(1):52–64. doi: 10.1016/j.gpb.2019.08.002 (PMC7393547; doi:10.1016/j.gpb.2019.08.002)
Supplement: Supplementary Table S5 [file mmc5.docx]

**Table S5 Statistics of proteome-wide substrate cleavage site predictions in 17,628 human 3D structures for 27 different proteases by Procleave**

| **Family** | **Protease** | **MEROPS ID** | **No. of predicted substrates** | **No. of predicted cleavage sites** |
| --- | --- | --- | --- | --- |
| Aspartic | Pepsin A | A01.001 | 17,342 | 272,976 |
|  | Cathepsin D | A01.009 | 552 | 1904 |
|  | Cathepsin E | A01.010 | 496 | 1735 |
|  | Rhizopuspepsin | A01.012 | 622 | 3546 |
|  | Aspergillopepsin I | A01.016 | 654 | 7595 |
|  | Necepsin-1 | A01.053 | 663 | 13,140 |
|  | HIV-1 retropepsin | A02.001 | 549 | 1526 |
| Cysteine | Cathepsin L | C01.032 | 582 | 3543 |
|  | Cathepsin L1 (*Fasciola* sp.) | C01.033 | 657 | 62,146 |
|  | Cathepsin S | C01.034 | 458 | 1556 |
|  | Falcipain-2 | C01.046 | 646 | 30,299 |
|  | Cathepsin B | C01.060 | 353 | 1234 |
|  | Falcipain-3 | C01.063 | 636 | 18,773 |
|  | Caspase-3 | C14.003 | 169 | 241 |
|  | Caspase-6 | C14.005 | 238 | 389 |
| Metallo | MMP-2 | M10.003 | 593 | 5101 |
|  | MMP-9 | M10.004 | 548 | 1960 |
|  | Astacin | M12.001 | 306 | 794 |
|  | Meprin alpha | M12.002 | 526 | 2184 |
|  | Meprin beta | M12.004 | 416 | 1173 |
|  | LAST_MAM peptidase | M12.033 | 520 | 2476 |
| Serine | Chymotrypsin A (bovine) | S01.001 | 557 | 3564 |
|  | Granzyme B (human) | S01.010 | 317 | 729 |
|  | Elastase-2 | S01.131 | 416 | 1646 |
|  | Cathepsin G | S01.133 | 225 | 445 |
|  | Glutamyl peptidase-1 | S01.269 | 479 | 1537 |
|  | Lysyl peptidase (bacteria) | S01.280 | 316 | 622 |

*Note*: We chose a high-confidence specificity level (Sp ≥ 99%) for each protease to generate high-confidence prediction results.
