# Supplementary material for: Procleave: Predicting Protease-specific Substrate Cleavage Sites by Combining Sequence and Structural Information
Source: Genomics Proteomics Bioinformatics. 2020 May 12;18(1):52–64. doi: 10.1016/j.gpb.2019.08.002 (PMC7393547; doi:10.1016/j.gpb.2019.08.002)
Supplement: Supplementary Table S6 [file mmc6.docx]

**Table S6**  **Summary of the predicted cleavage sites by Procleave for the six substrate structures in the case study**

| **Protein name (PDB ID) of the substrate** | **Chain** | **Ranking** | **Cleavage position** | **Cleavage site (P4–P4′)** | **Cleaved by protease** | **Cleavage probability score** |
| --- | --- | --- | --- | --- | --- | --- |
| Human alphaB crystalline (3L1G) | A | 1 | **88** | SPEE \| LKVK | MMP-9 | 0.966 |
|  | A | 2 | **150** | GPRK \| QVSG |  | 0.966 |
|  | A | 3 | **132** | DPLT \| ITSS |  | 0.953 |
|  | A | 4 | **99** | DVIE \| VHGK |  | 0.937 |
|  | A | 5 | **78** | FSVN \| LDVK |  | 0.923 |
|  | A | 6 | **136** | ITSS \| LSSD |  | 0.920 |
|  | A | 7 | **102** | EVHG \| KHEE |  | 0.916 |
|  | A | 8 | **121** | FHRK \| YRIP |  | 0.915 |
|  | A | 9 | **112** | DEHG \| FISR |  | 0.884 |
|  | A | 10 | **100** | VIEV \| HGKH |  | 0.844 |
| Human interferon beta (1AU1) | A | 1 | **4** | MSYN \| LLGF | MMP-9 | 0.971 |
|  | A | 2 | **86** | IVEN \| LLAN |  | 0.959 |
|  | A | 3 | **8** | LLGF \| LQRS |  | 0.935 |
|  | A | 4 | **93** | NVYH \| QINH |  | 0.916 |
|  | A | 5 | **9** | LGFL \| QRSS |  | 0.905 |
| ATPase p97 mutant (3HU2) | A | 1 | 169 | VETD \| PSPY | Caspase-6 | 0.950 |
|  | A | 2 | **204** | VGYD \| DIGG |  | 0.947 |
|  | A | 3 | **450** | VTMD \| DFRW |  | 0.943 |
|  | A | 4 | **307** | DELD \| AIAP |  | 0.940 |
|  | A | 5 | **368** | REVD \| IGIP |  | 0.908 |
|  | A | 6 | **410** | VGAD \| LAAL |  | 0.887 |
|  | A | 7 | **395** | DDVD \| LEQV |  | 0.848 |
| Human enolase 1 (3B97) | A | 1 | 220 | LENK \| EGLE | Meprin beta | 0.958 |
|  | A | 2 | **165** | LAMQ \| EFMI |  | 0.953 |
|  | A | 3 | 292 | VSIE \| DPFD |  | 0.940 |
|  | A | 4 | **139** | LAGN \| SEVI |  | 0.931 |
|  | A | 5 | 284 | SFIK \| DYPV |  | 0.920 |
|  | A | 6 | 381 | TFIA \| DLVV |  | 0.877 |
|  | A | 7 | **207** | TNVG \| DEGG |  | 0.865 |
|  | A | 8 | **140** | AGNS \| EVIL |  | 0.851 |

*Note*: All the predicted cleavage sites are listed, and experimentally verified cleavage sites are put in bold. The cleavage probability score was generated by the CRF models of Procleave. The higher the cleavage score, the more likely a cleavage site is predicted to be cleaved. Here, “|” indicates the substrate cleavage site after the P1 position.
