# Supplementary figures and images for: Procleave: Predicting Protease-specific Substrate Cleavage Sites by Combining Sequence and Structural Information
Source: Genomics Proteomics Bioinformatics. 2020 May 12;18(1):52–64. doi: 10.1016/j.gpb.2019.08.002 (PMC7393547; doi:10.1016/j.gpb.2019.08.002)

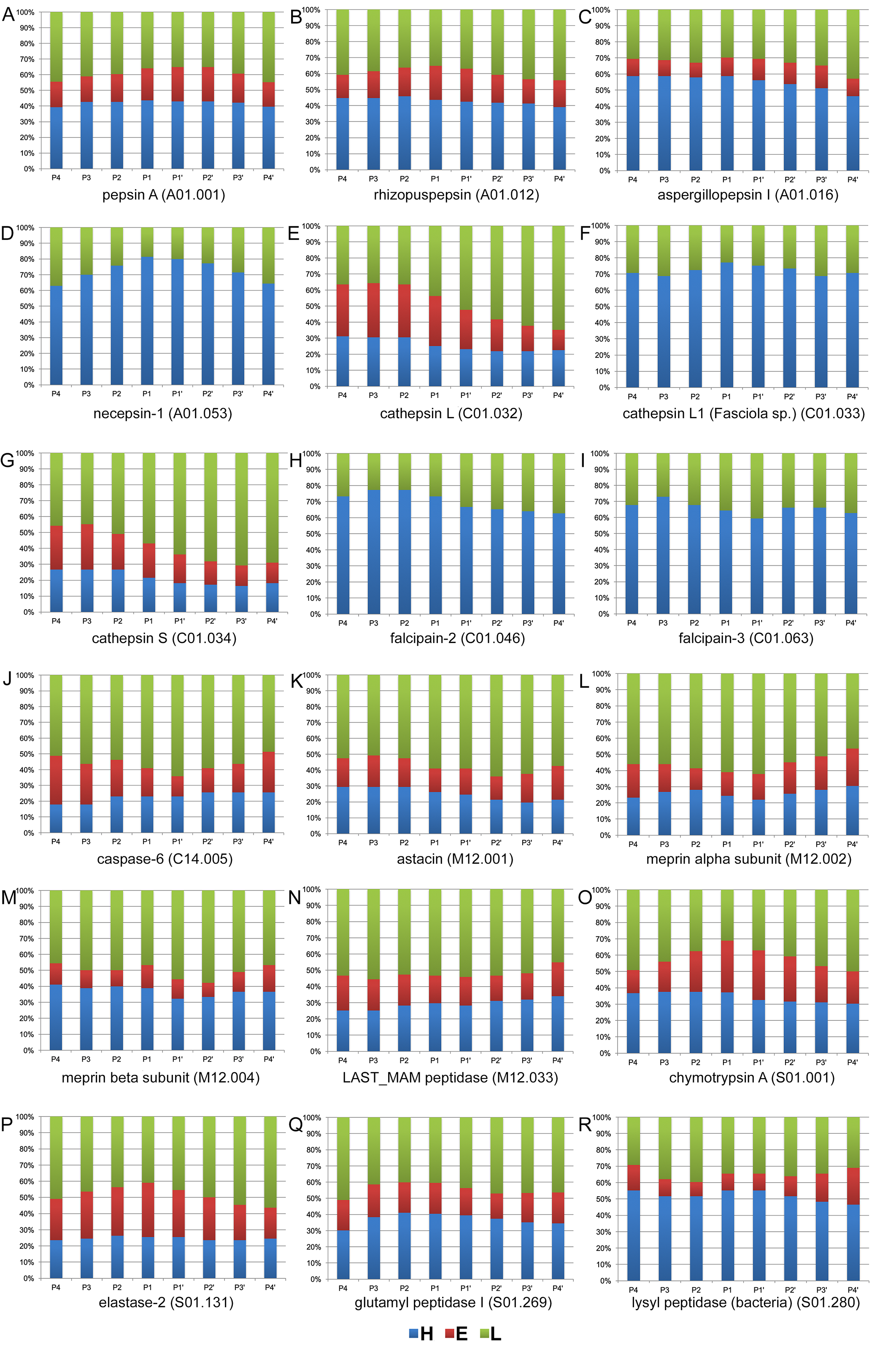

Supplement: Supplementary Figure S1 — Structural determinants of the substrate specificity of 18 proteases across the P4–P4′ cleavage sites. A. Pepsin A. B. Rhizopuspepsin. C. Aspergillopepsin. D. Necepsin-1. E. Cathepsin L. F. Cathepsin L1 (Fasciola sp.). G. Cathepsin S. H. Falcipain-2. I. Falcipain-3. J. Caspase-6. K. Astacin. L. Meprin alpha subunit. M. Meprin beta subunit. N. LAST_MAM peptidase. O. Chymotrypsin A. P. Elastase-2. Q. Glutamyl peptidase I. R. Lysyl peptidase (bacteria). H, helix; E, strand; L, loop. [file mmc7.zip › Figure S1.png]

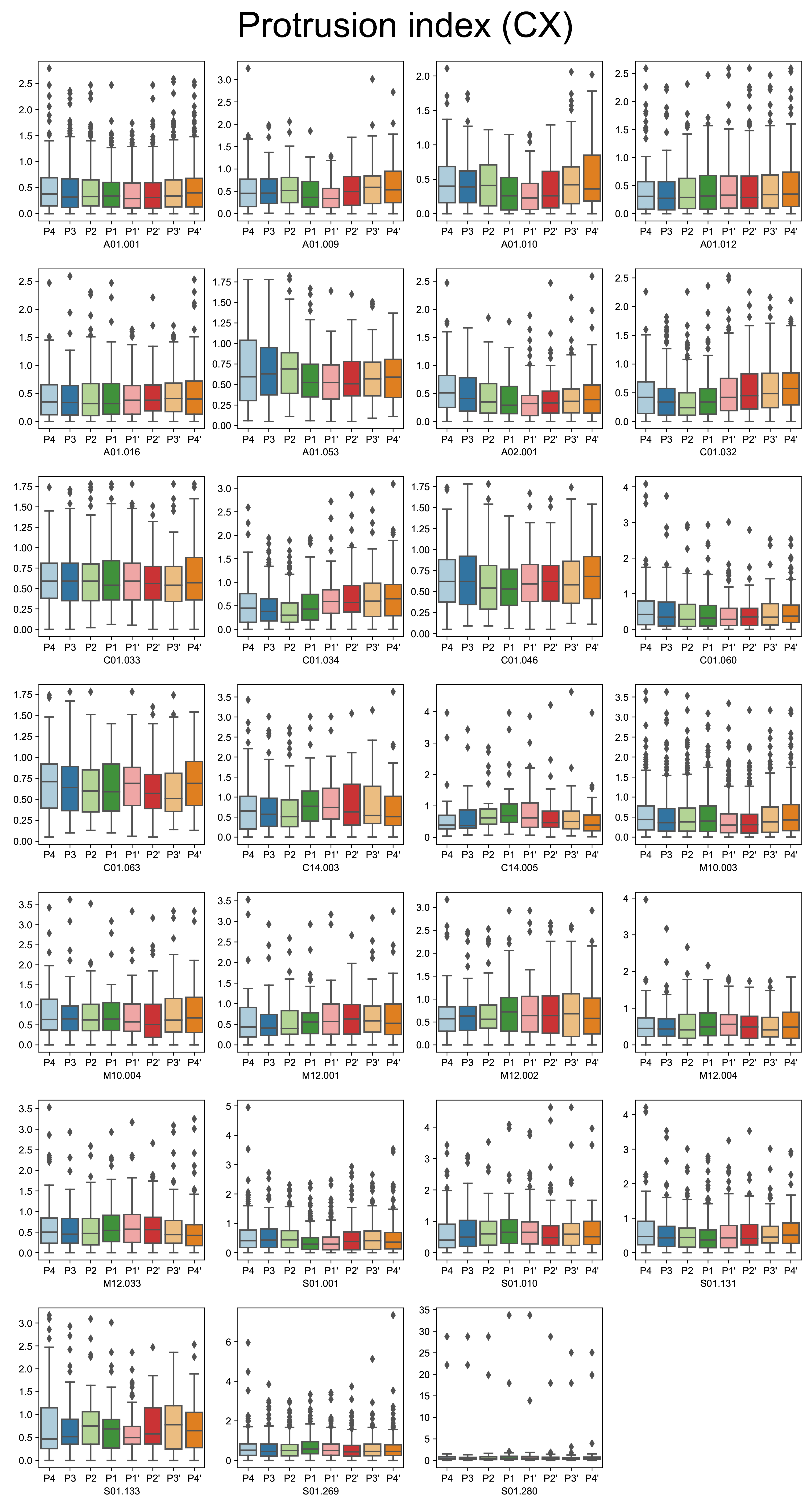

Supplement: Supplementary Figure S2 — Boxplots of protrusion index calculated by CX. [file mmc8.zip › Figure S2.png]

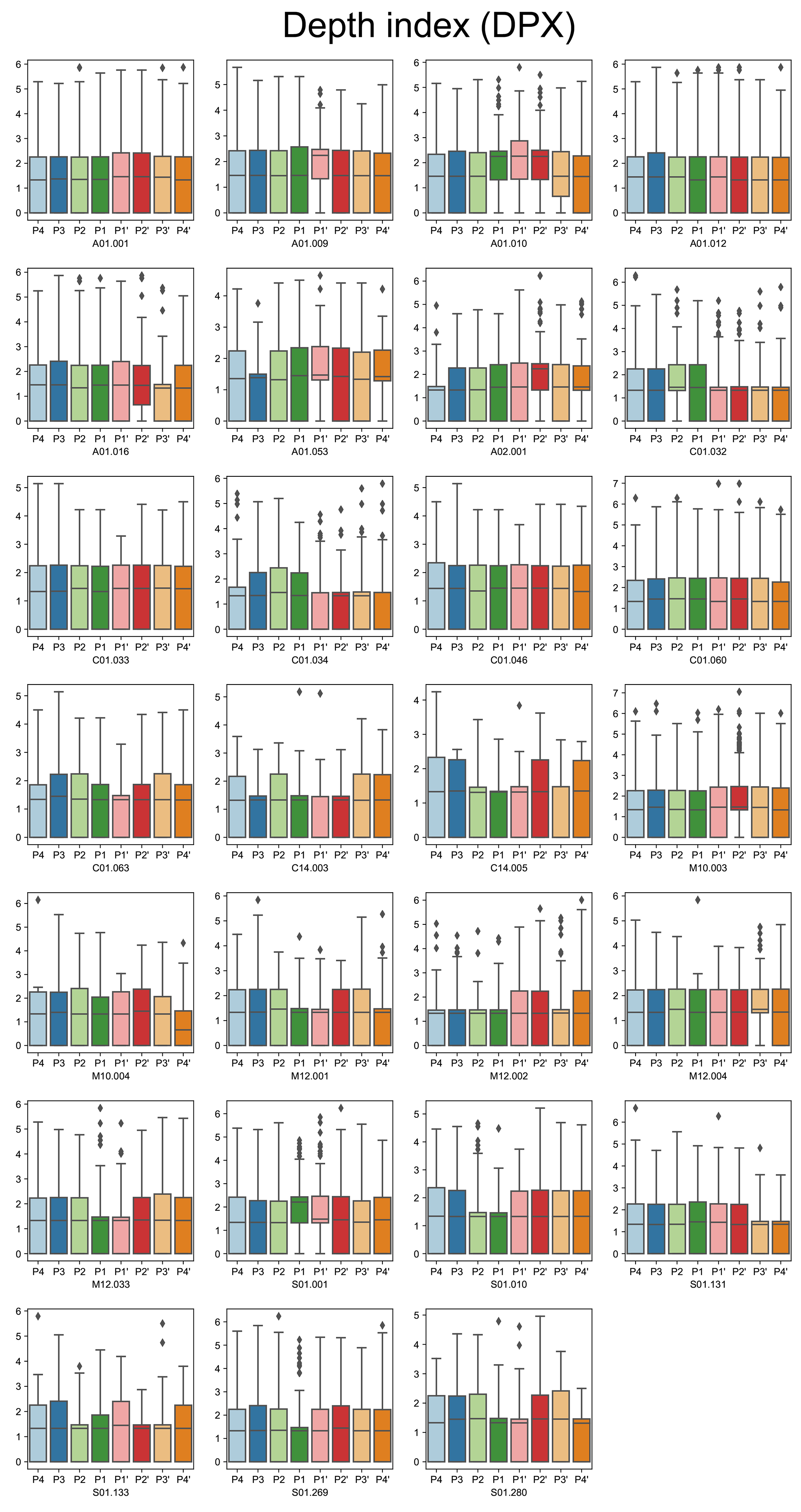

Supplement: Supplementary Figure S3 — Boxplots of depth index calculated by DPX. [file mmc9.zip › Figure S3.png]

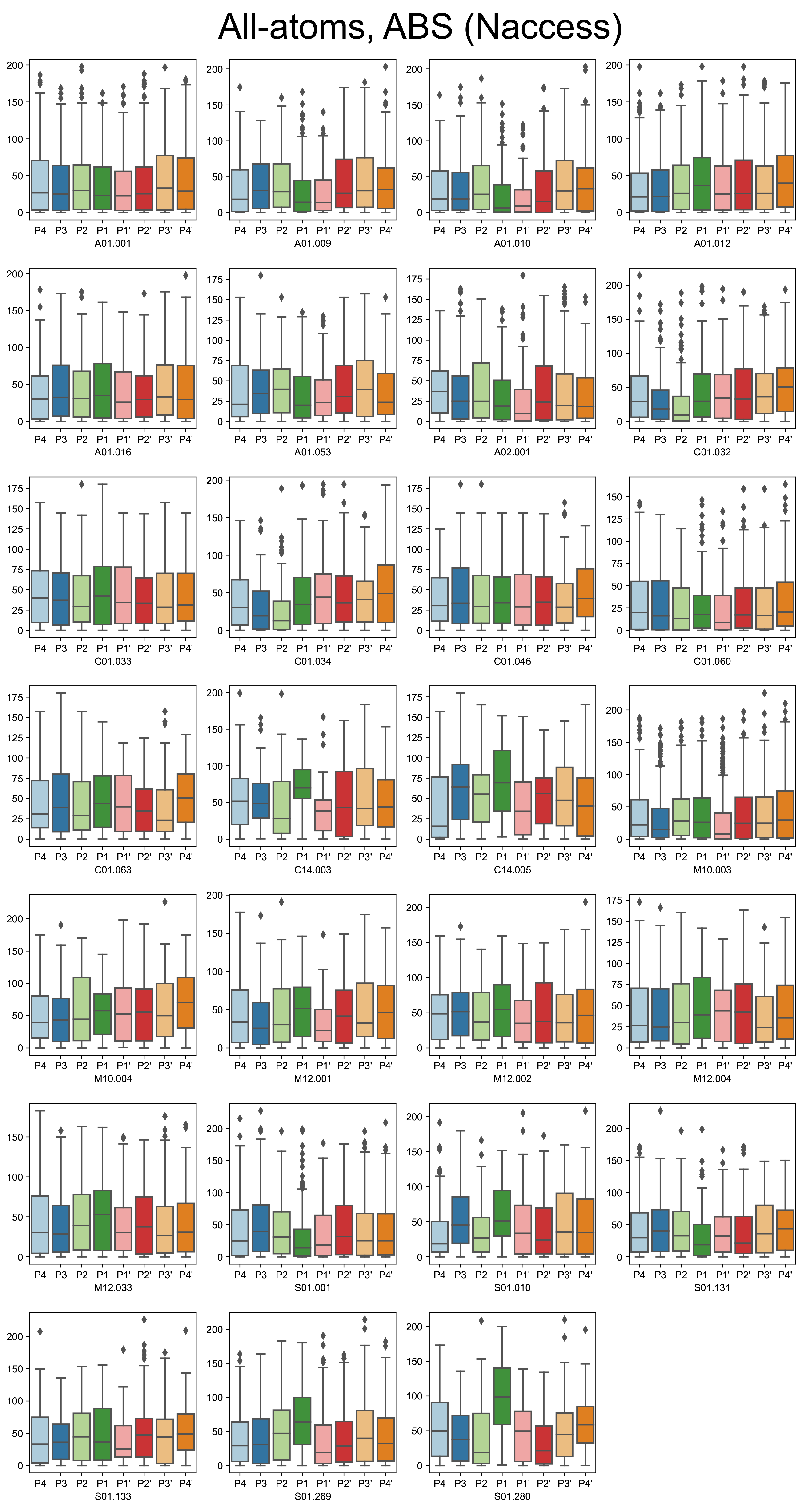

Supplement: Supplementary Figure S4 — Boxplots of all-atom absolute solvent accessibility calculated by Naccess. [file mmc10.zip › Figure S4.png]

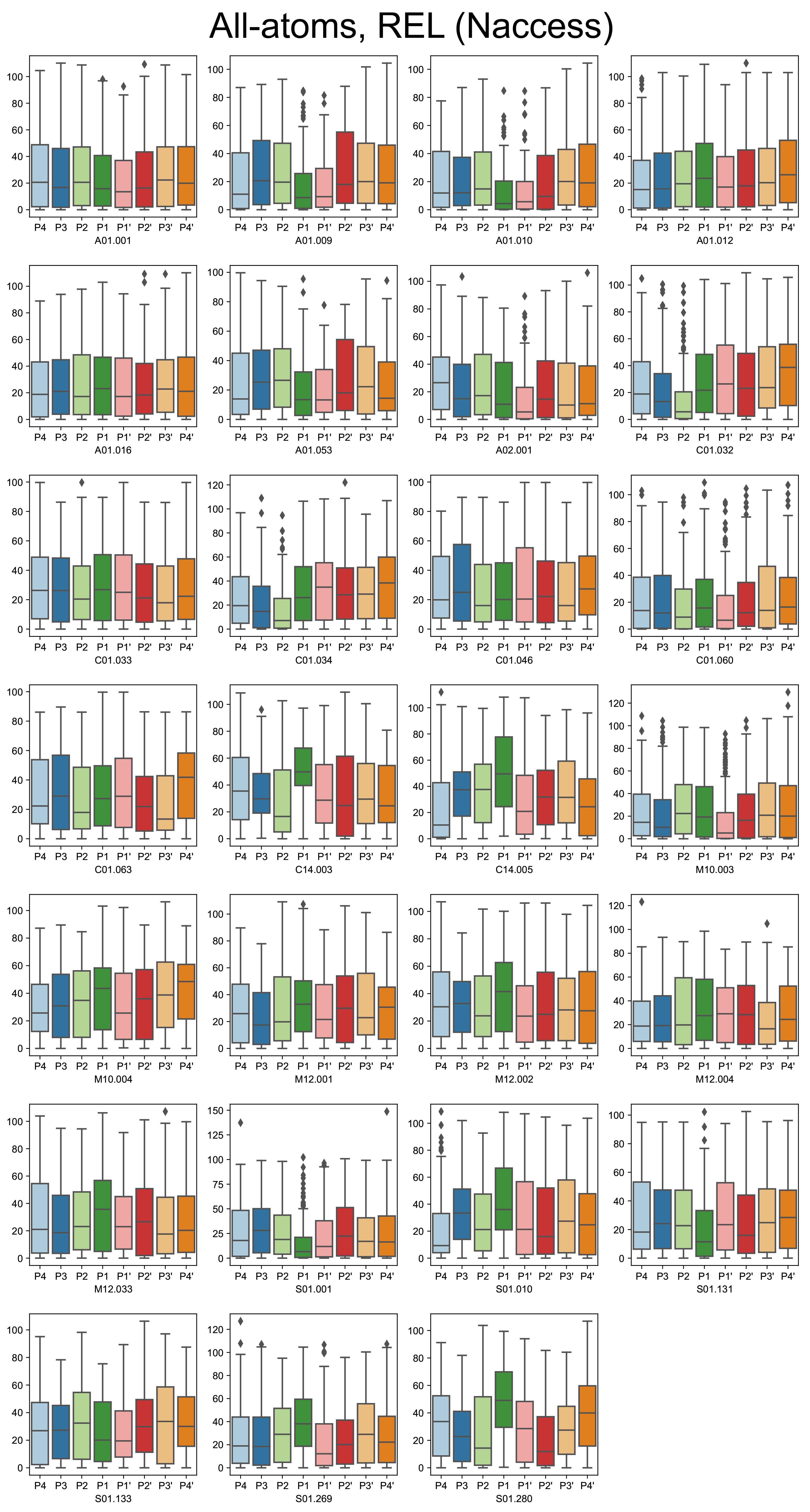

Supplement: Supplementary Figure S5 — Boxplots of all-atom relative solvent accessibility calculated by Naccess. [file mmc11.zip › Figure S5.png]

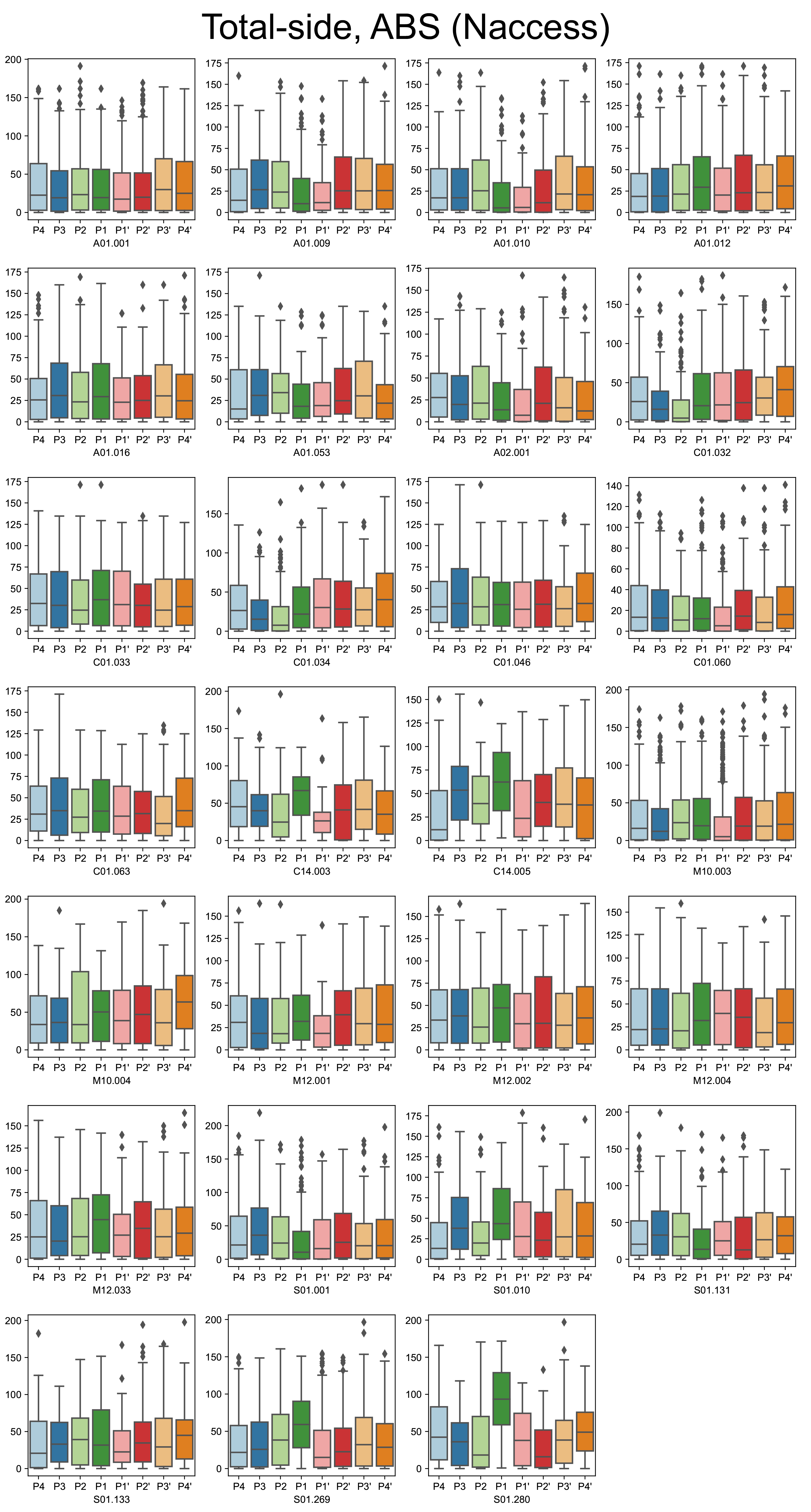

Supplement: Supplementary Figure S6 — Boxplots of total-side absolute solvent accessibility calculated by Naccess. [file mmc12.zip › Figure S6.png]

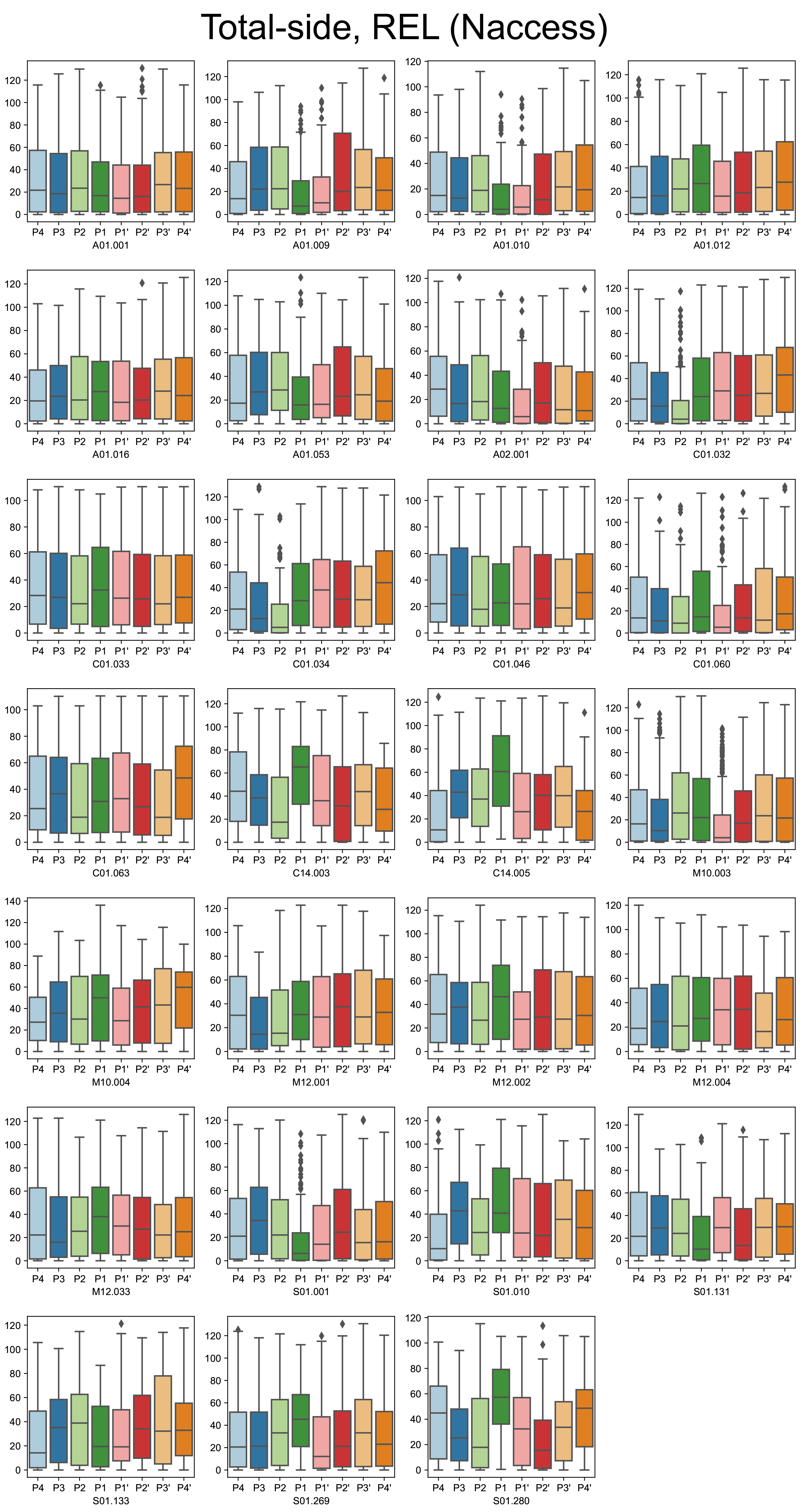

Supplement: Supplementary Figure S7 — Boxplots of total-side relative solvent accessibility calculated by Naccess. [file mmc13.zip › Figure S7.png]

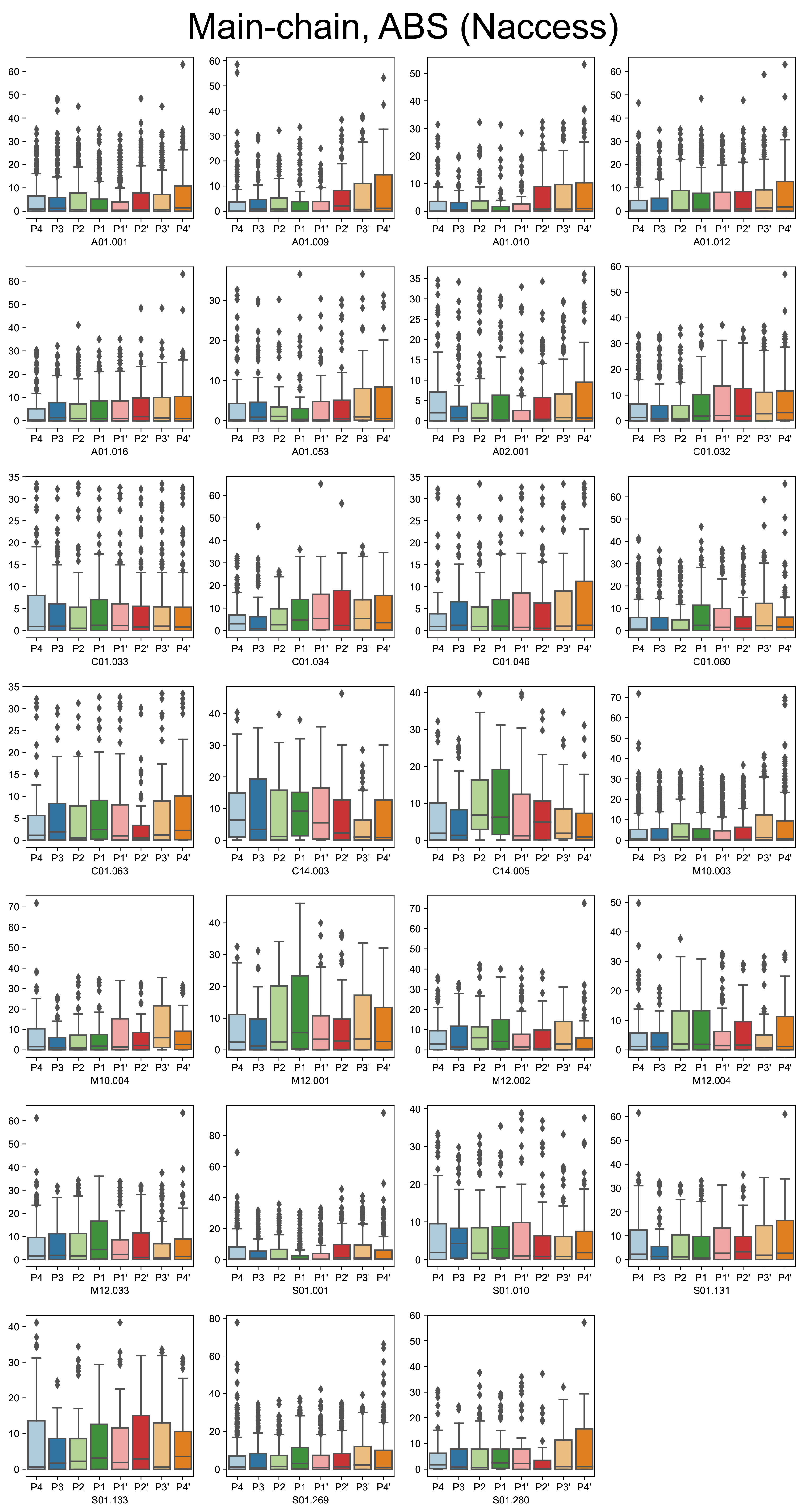

Supplement: Supplementary Figure S8 — Boxplots of main-chain absolute solvent accessibility calculated by Naccess. [file mmc14.zip › Figure S8.png]

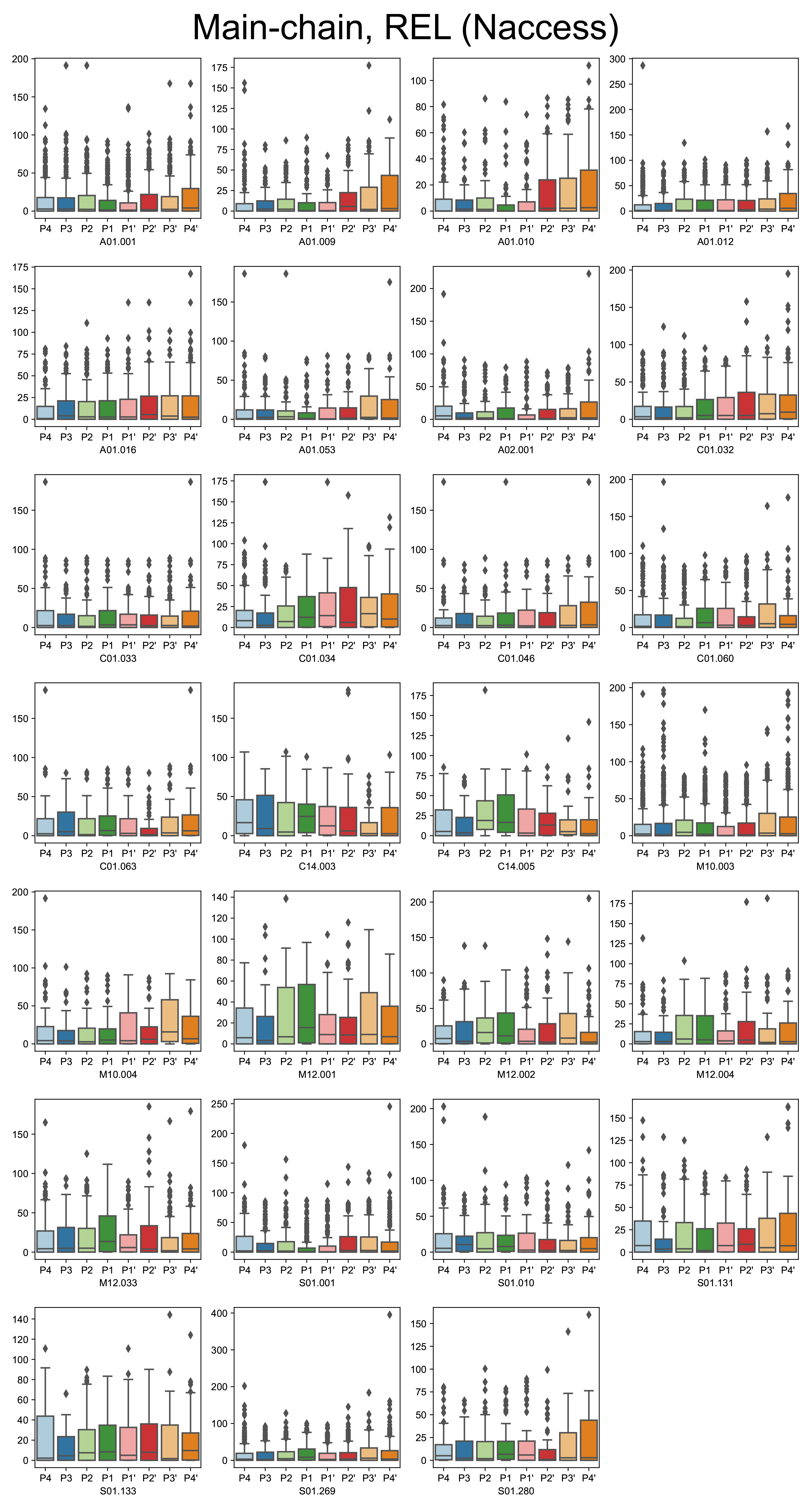

Supplement: Supplementary Figure S9 — Boxplots of main-chain relative solvent accessibility calculated by Naccess. [file mmc15.zip › Figure S9.png]

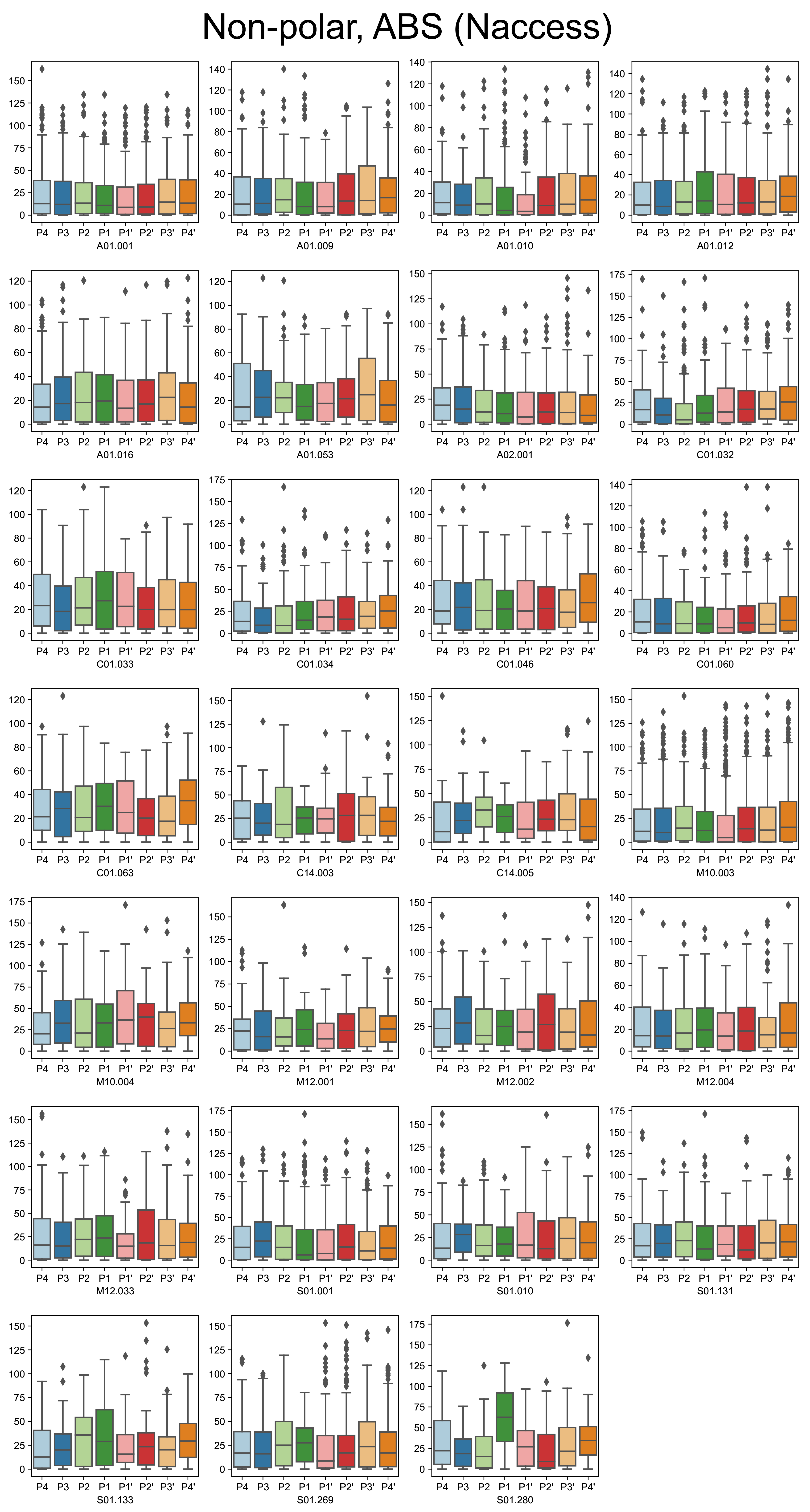

Supplement: Supplementary Figure S10 — Boxplots of non-polar absolute solvent accessibility calculated by Naccess. [file mmc16.zip › Figure S10.png]

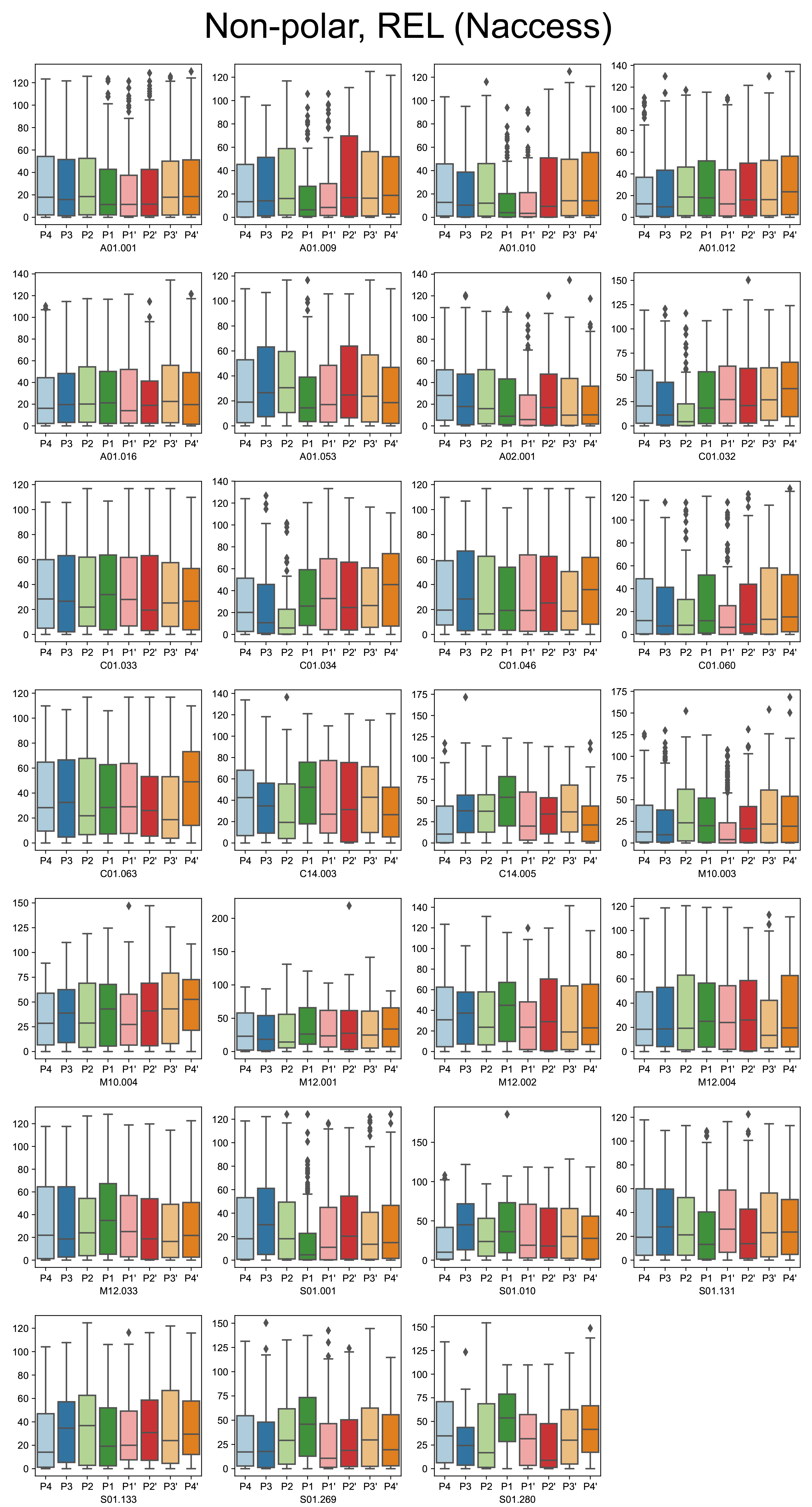

Supplement: Supplementary Figure S11 — Boxplots of non-polar relative solvent accessibility calculated by Naccess. [file mmc17.zip › Figure S11.png]

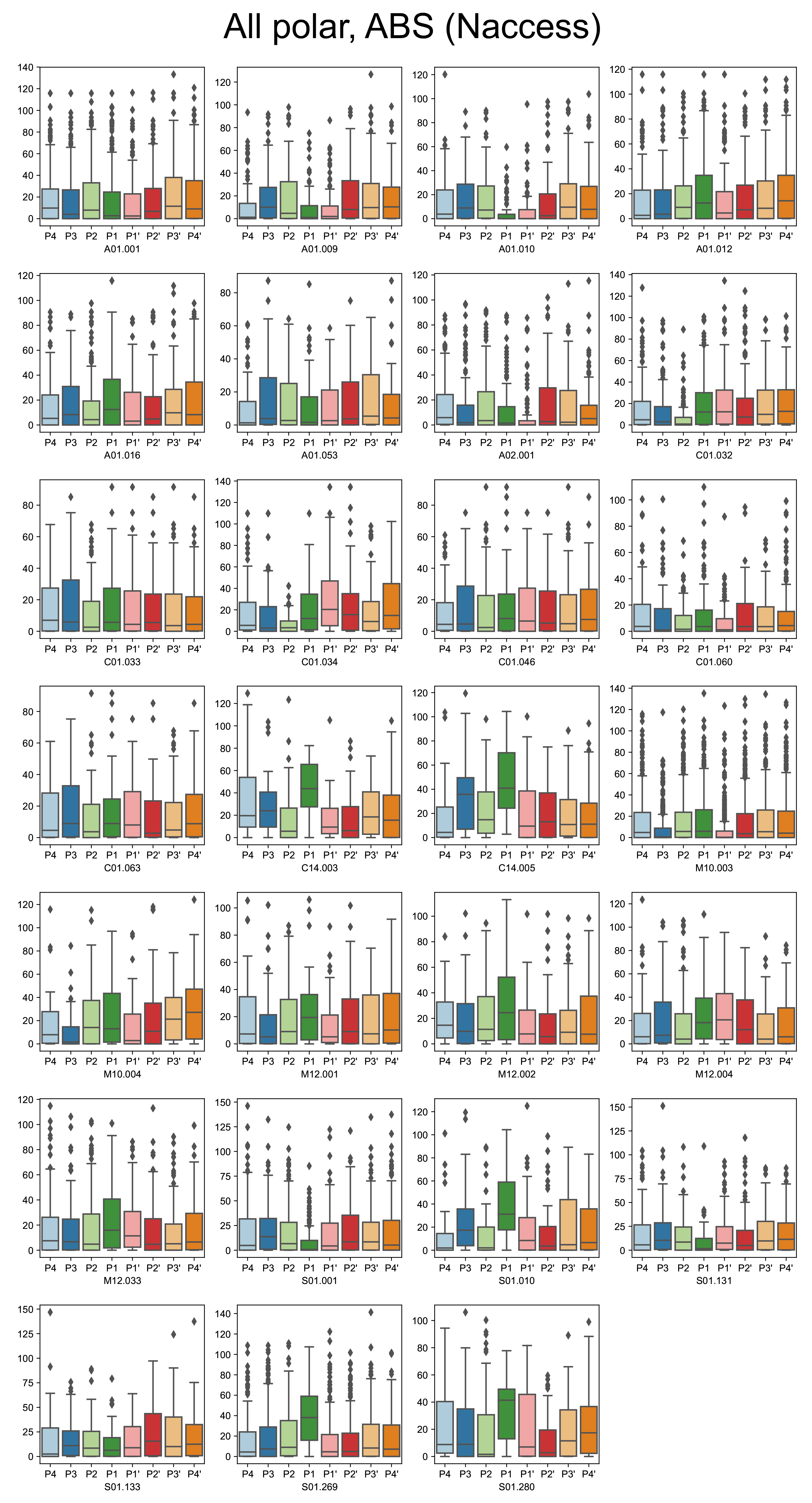

Supplement: Supplementary Figure S12 — Boxplots of all polar absolute solvent accessibility calculated by Naccess. [file mmc18.zip › Figure S12.png]

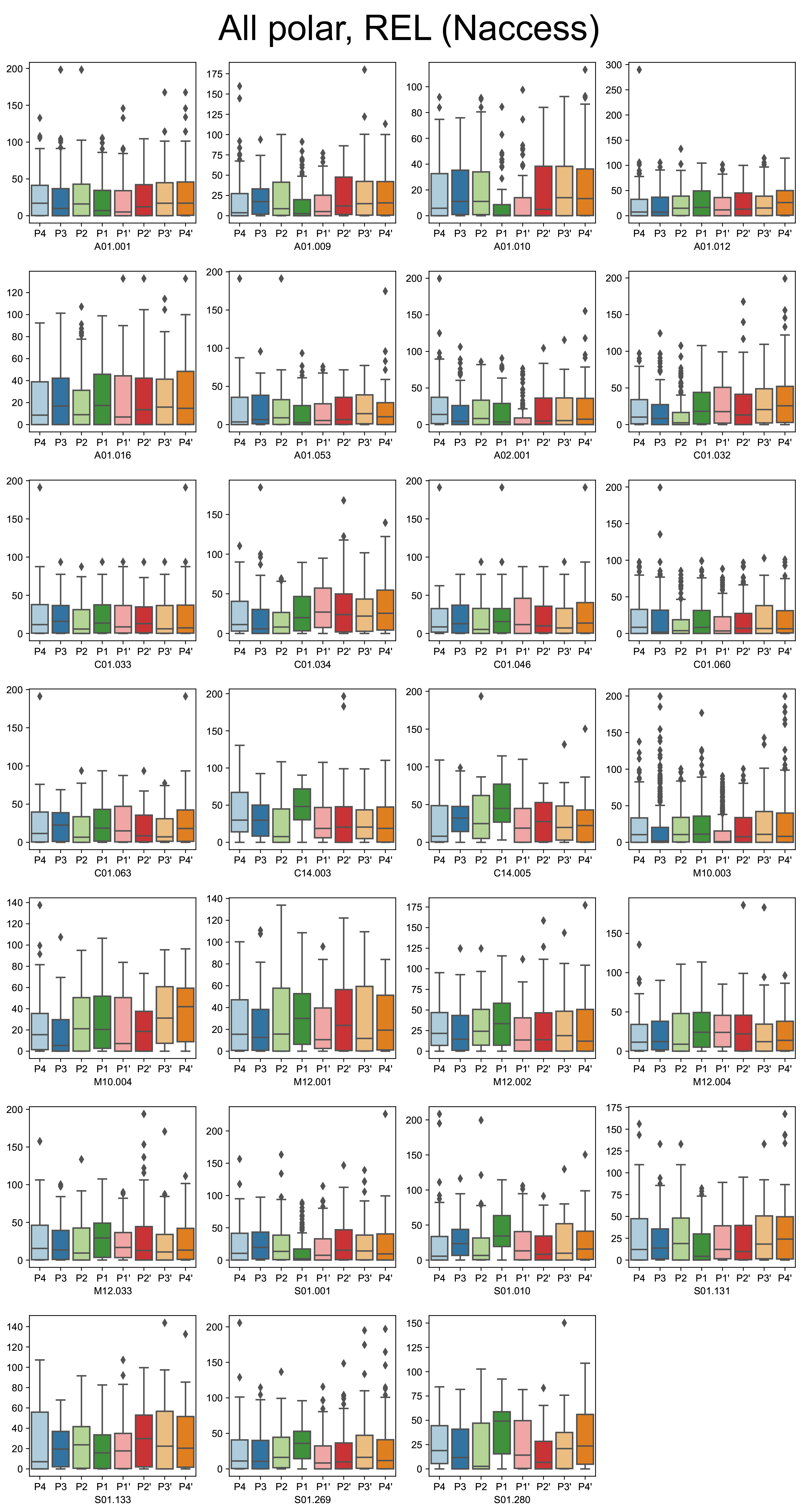

Supplement: Supplementary Figure S13 — Boxplots of all polar relative solvent accessibility calculated by Naccess. [file mmc19.zip › Figure S13.png]

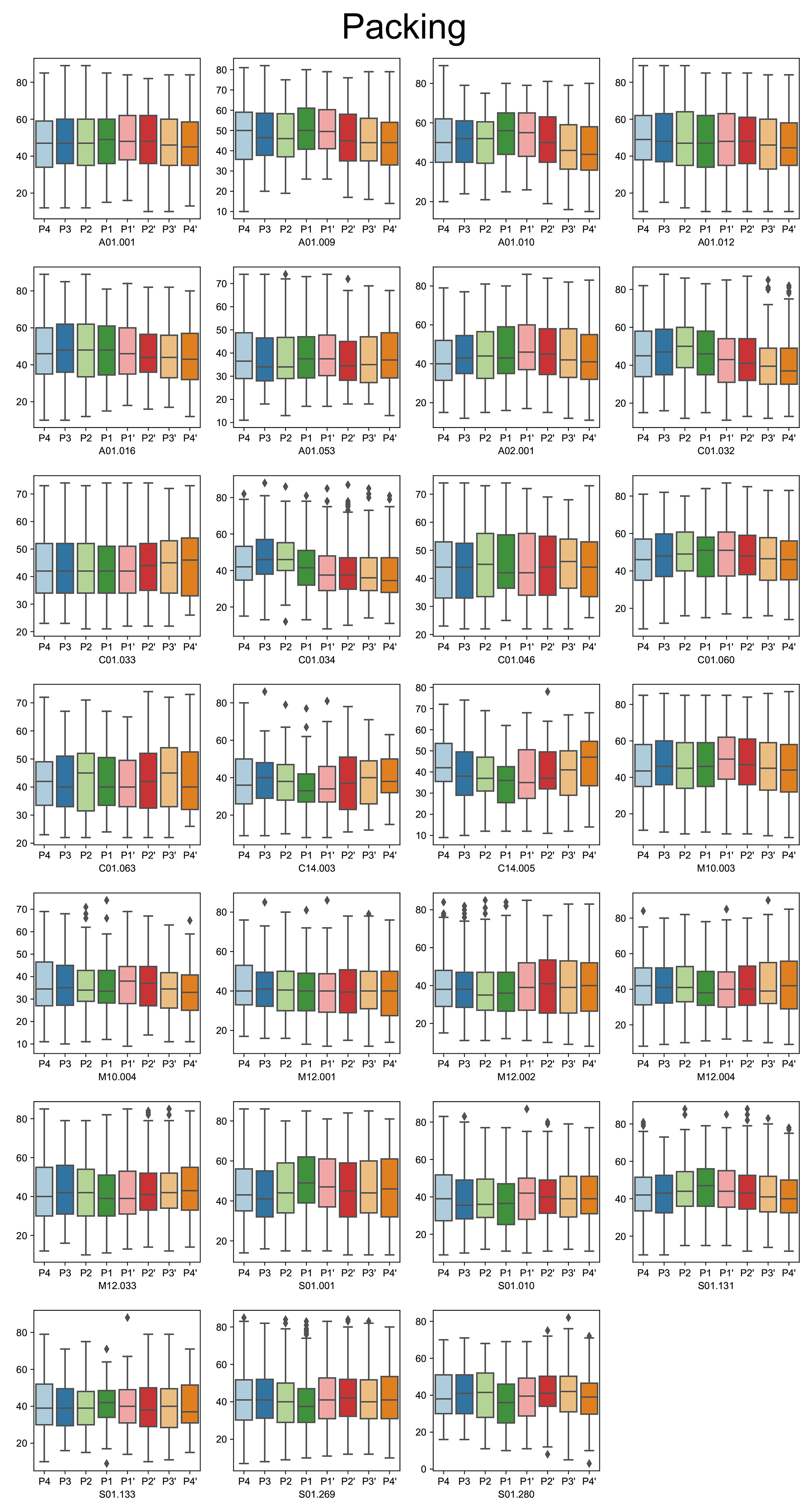

Supplement: Supplementary Figure S14 — Boxplots of packing feature. [file mmc20.zip › Figure S14.png]

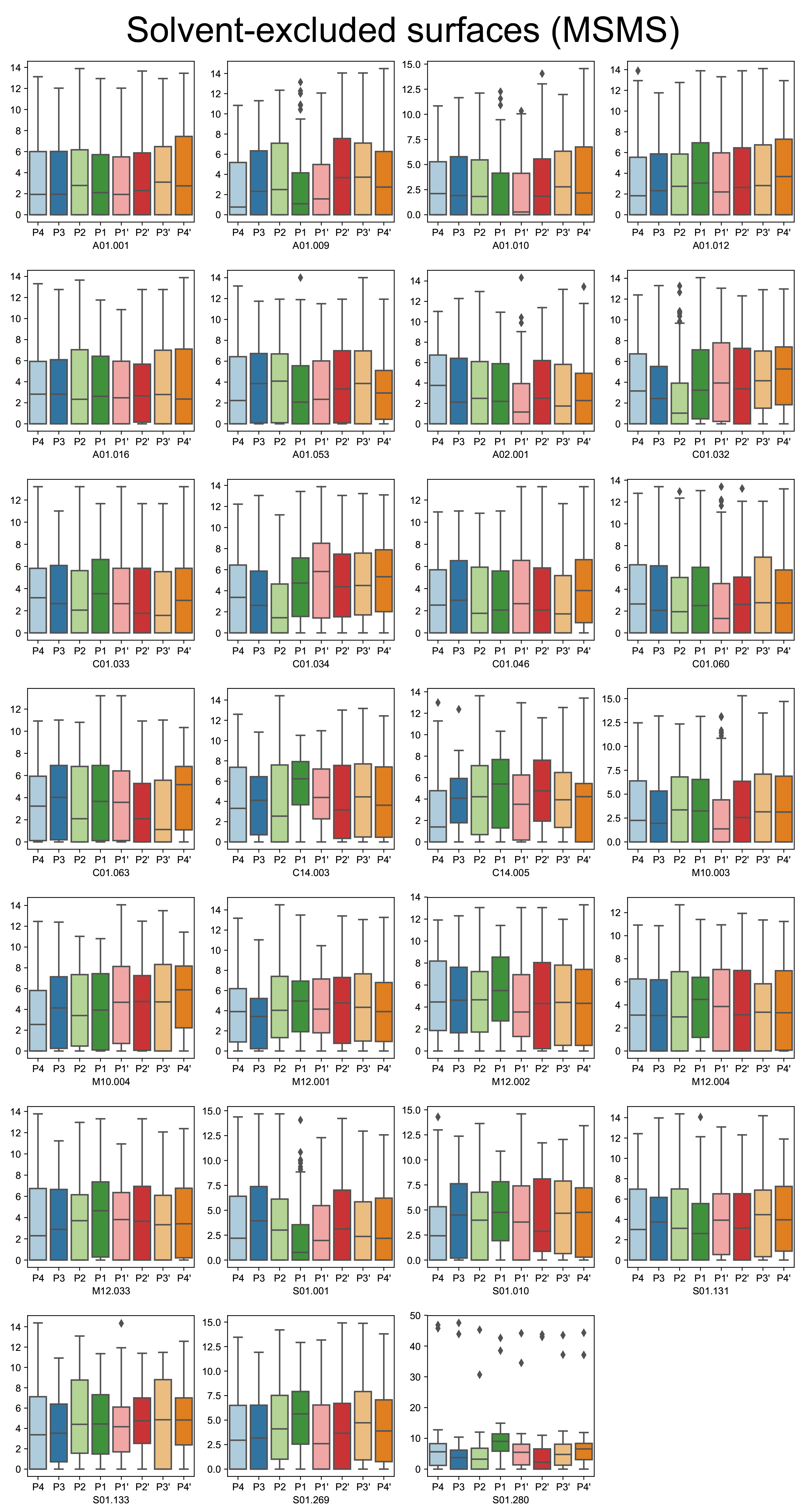

Supplement: Supplementary Figure S15 — Boxplots of molecular solvent-excluded surfaces calculated by MSMS. [file mmc21.zip › Figure S15.png]

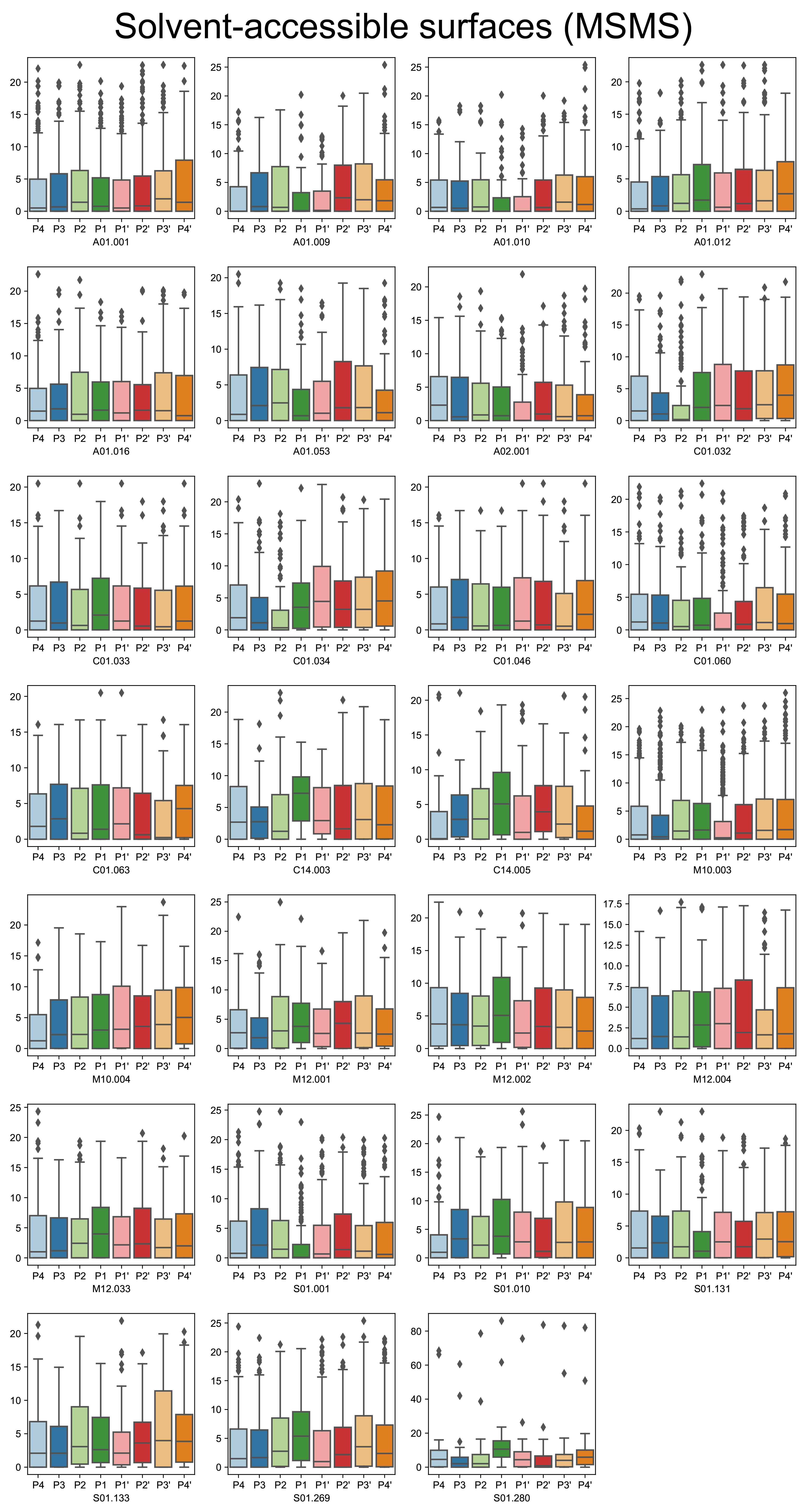

Supplement: Supplementary Figure S16 — Boxplots of molecular solvent-accessible surfaces calculated by MSMS. [file mmc22.zip › Figure S16.png]

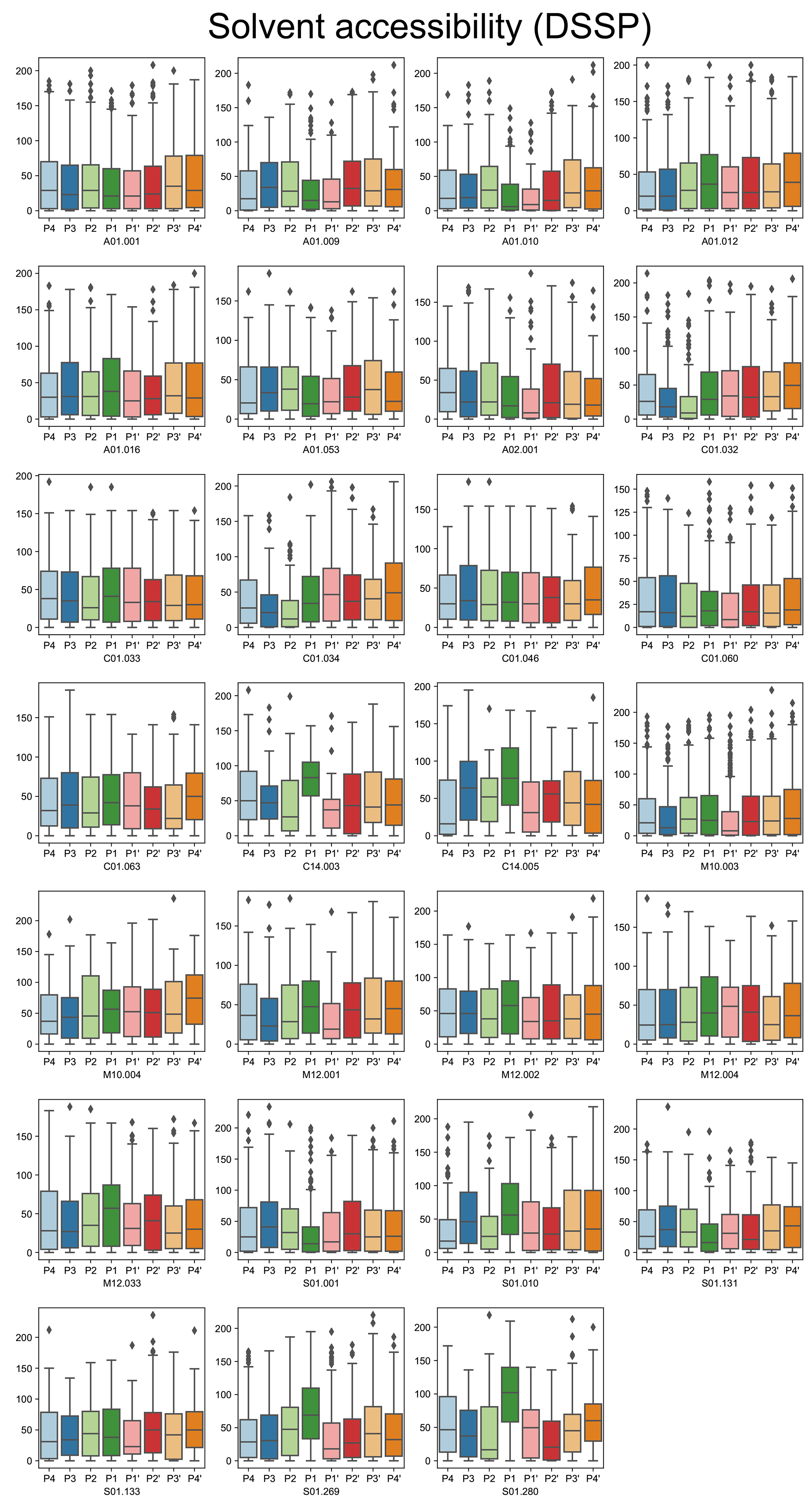

Supplement: Supplementary Figure S17 — Boxplots of solvent accessibility calculated by DSSP. [file mmc23.zip › Figure S17.png]

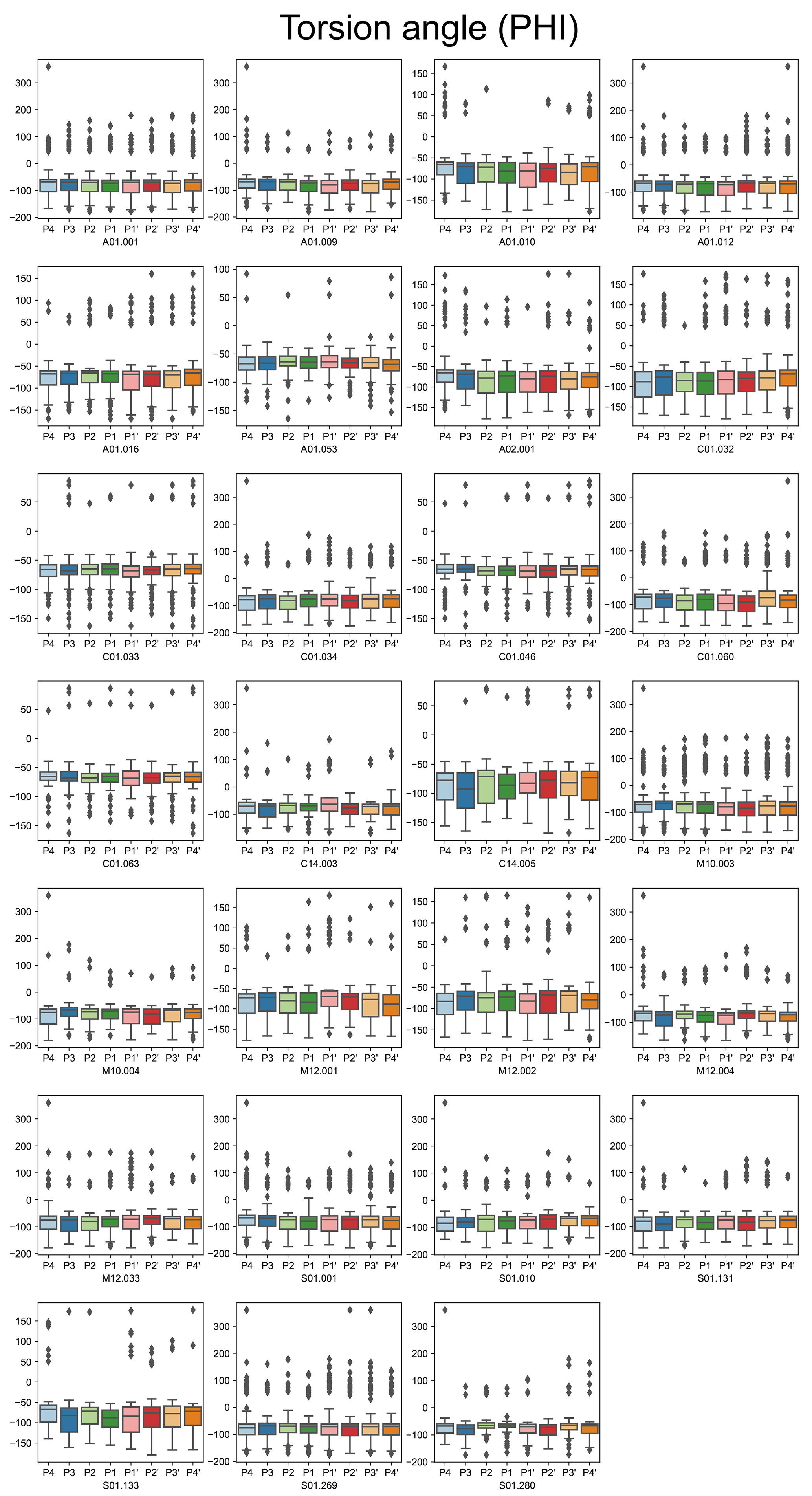

Supplement: Supplementary Figure S18 — Boxplots of torsion angle (PHI) calculated by DSSP. [file mmc24.zip › Figure S18.png]

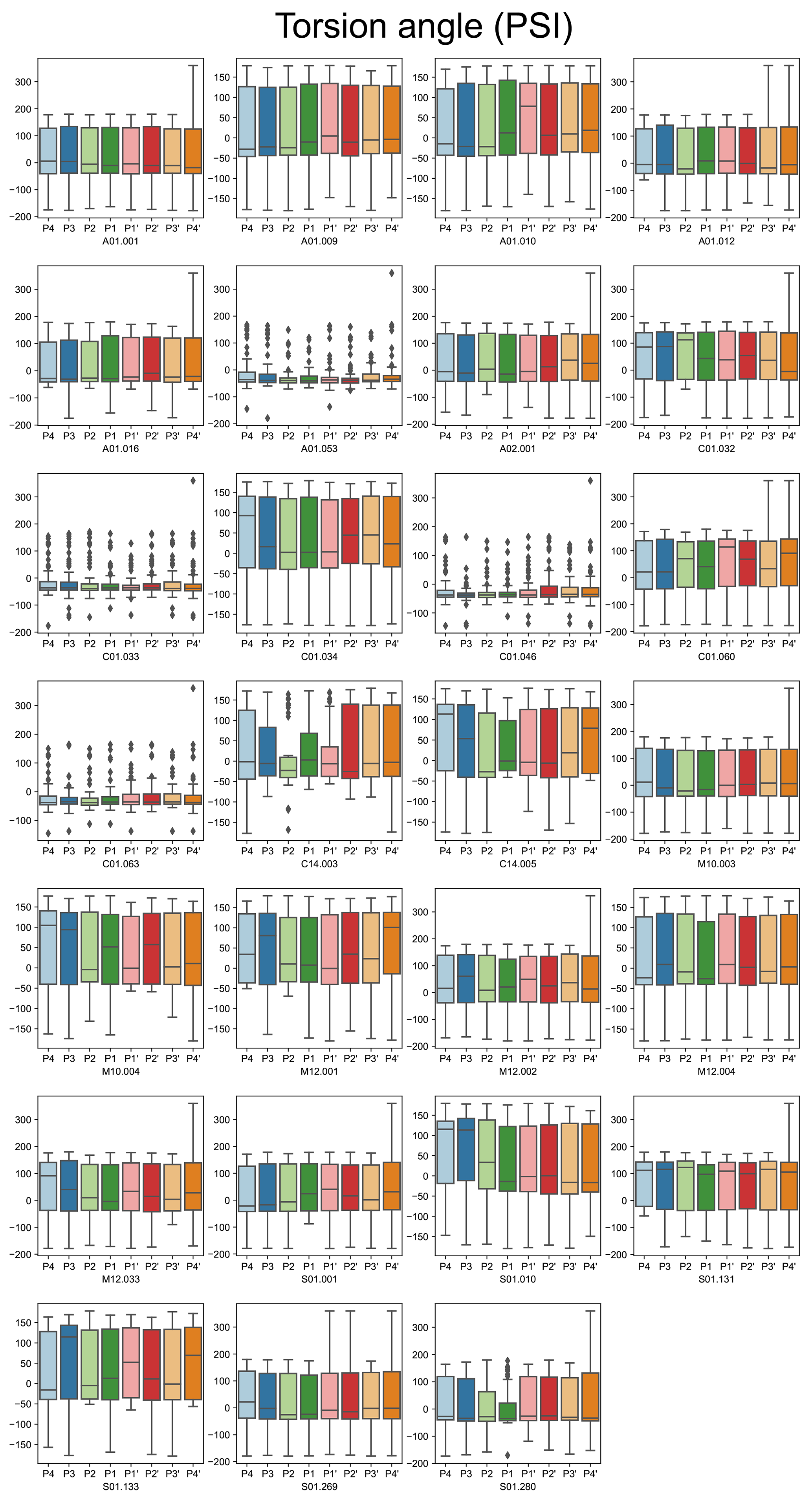

Supplement: Supplementary Figure S19 — Boxplots of torsion angle (PSI) calculated by DSSP. [file mmc25.zip › Figure S19.png]

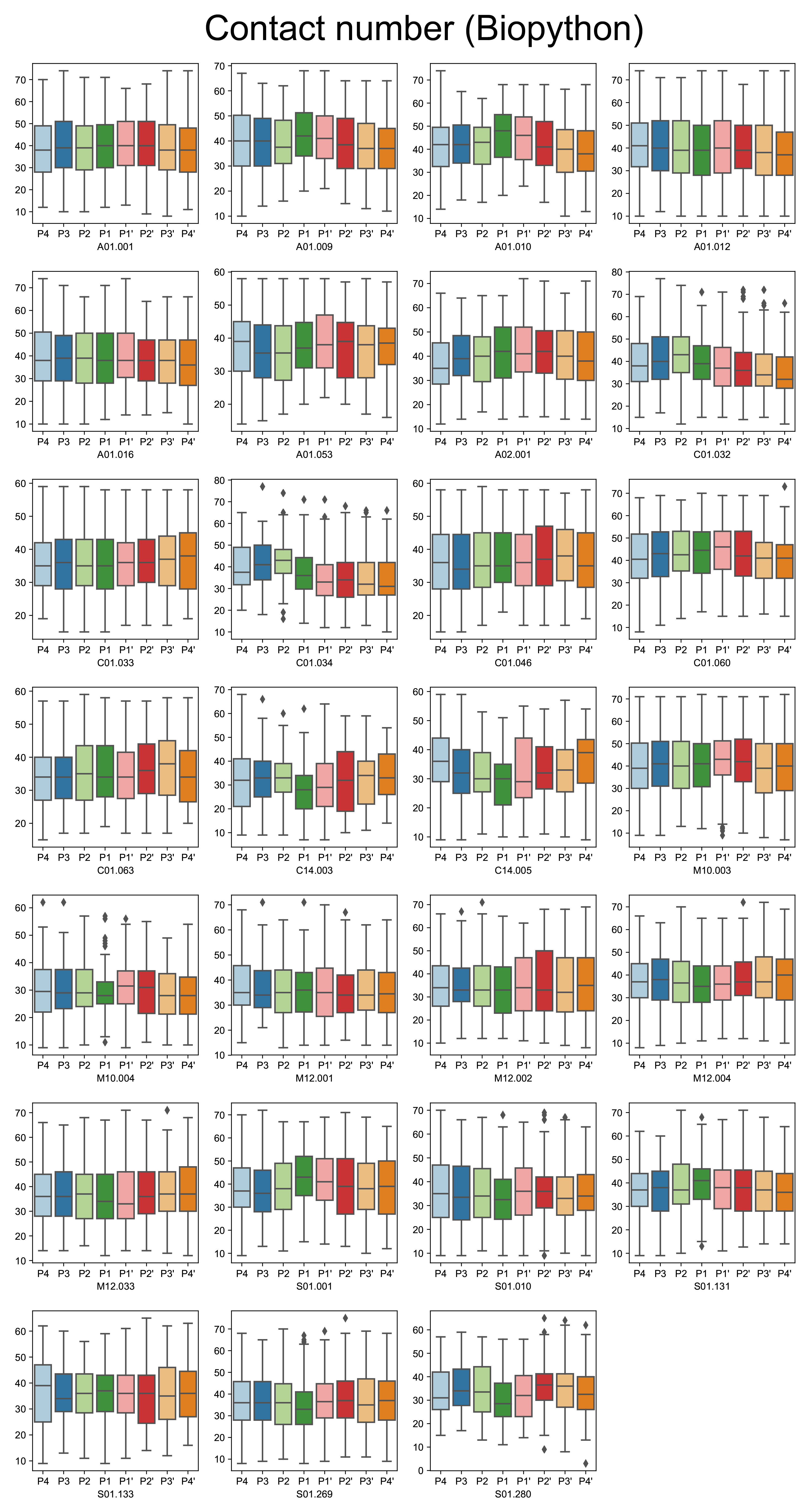

Supplement: Supplementary Figure S20 — Boxplots of contact number calculated by Biopython. [file mmc26.zip › Figure S20.png]

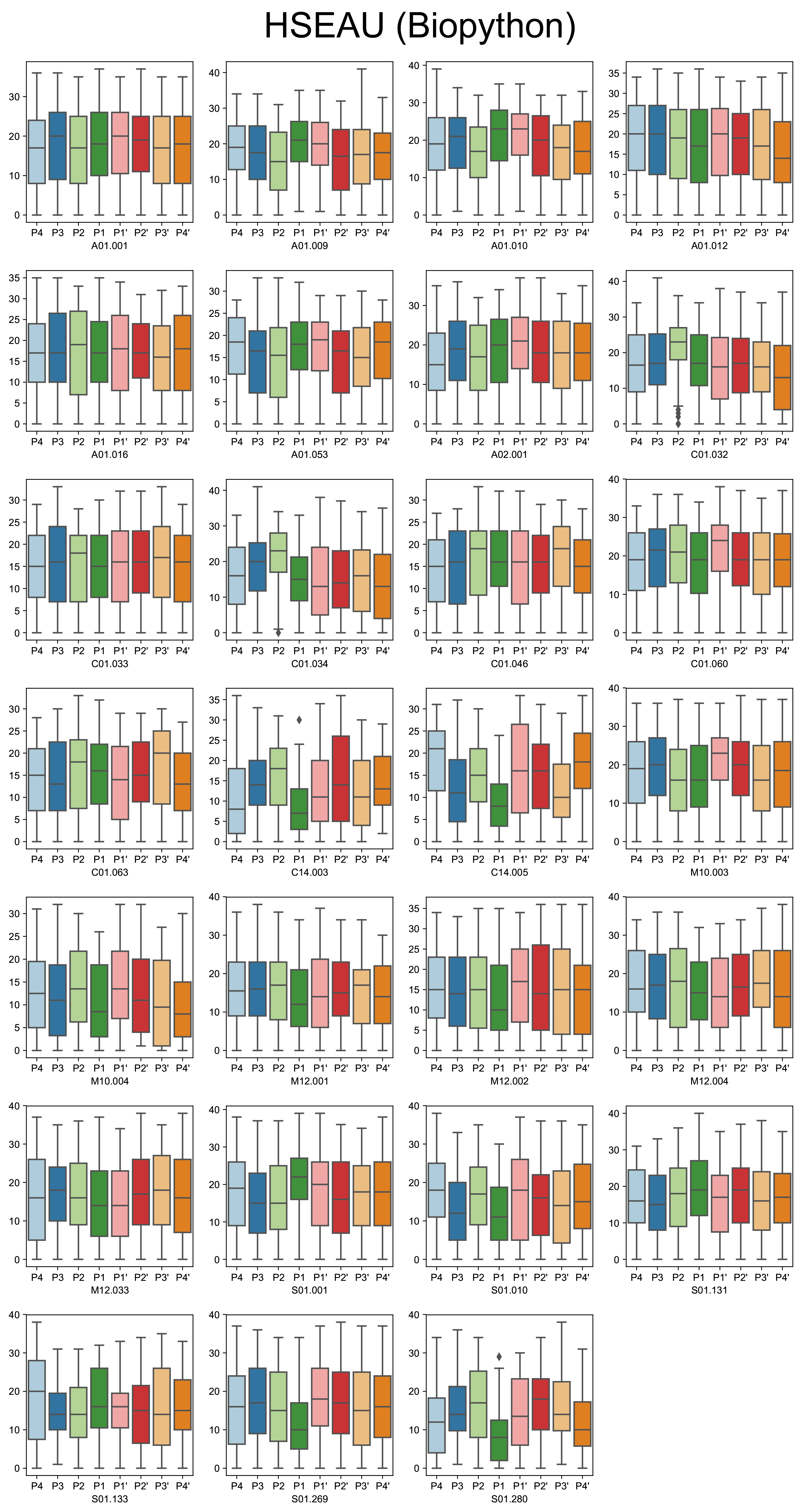

Supplement: Supplementary Figure S21 — Boxplots of HSEAU calculated by Biopython. [file mmc27.zip › Figure S21.png]

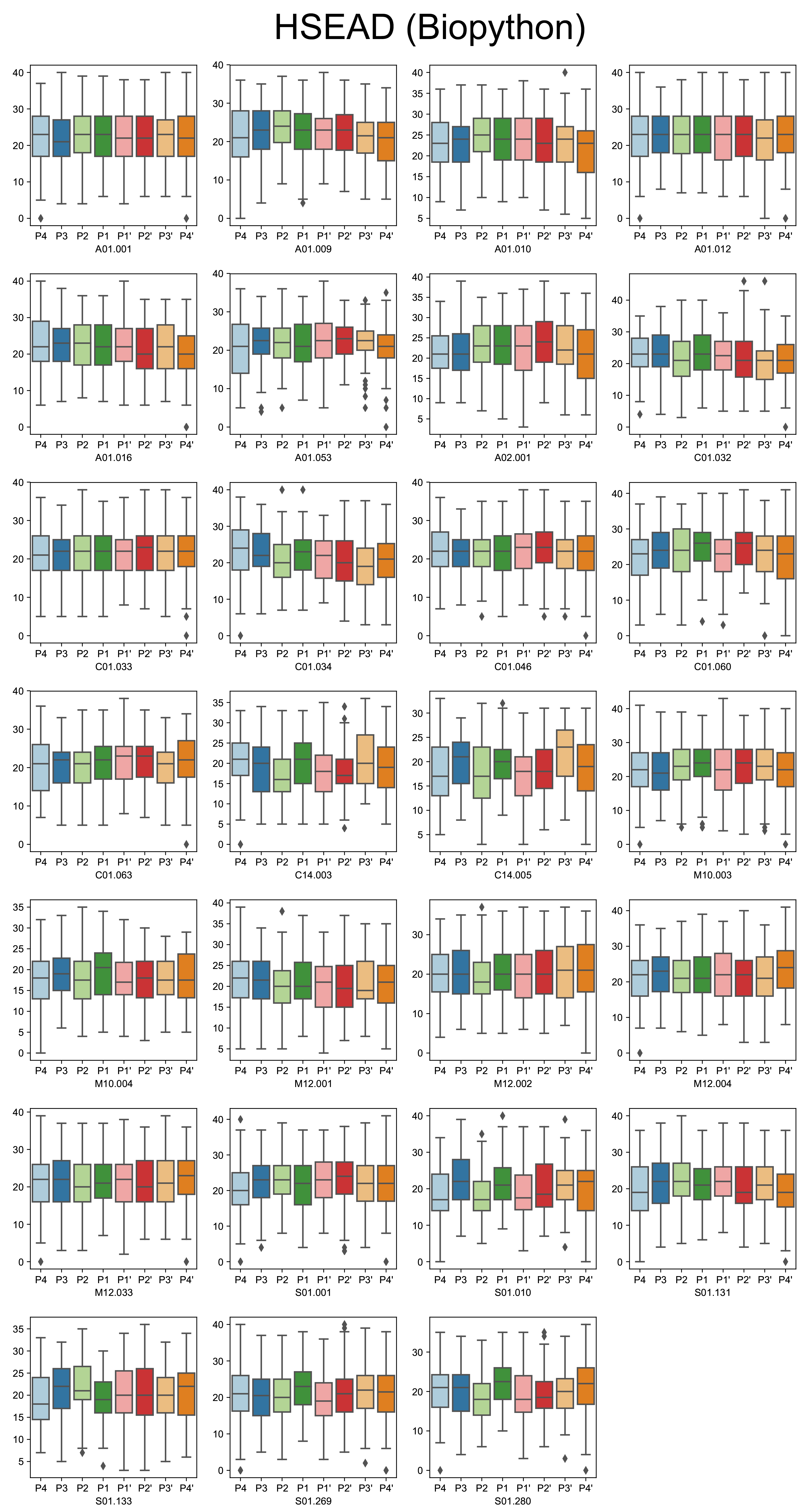

Supplement: Supplementary Figure S22 — Boxplots of HSEAD calculated by Biopython. [file mmc28.zip › Figure S22.png]

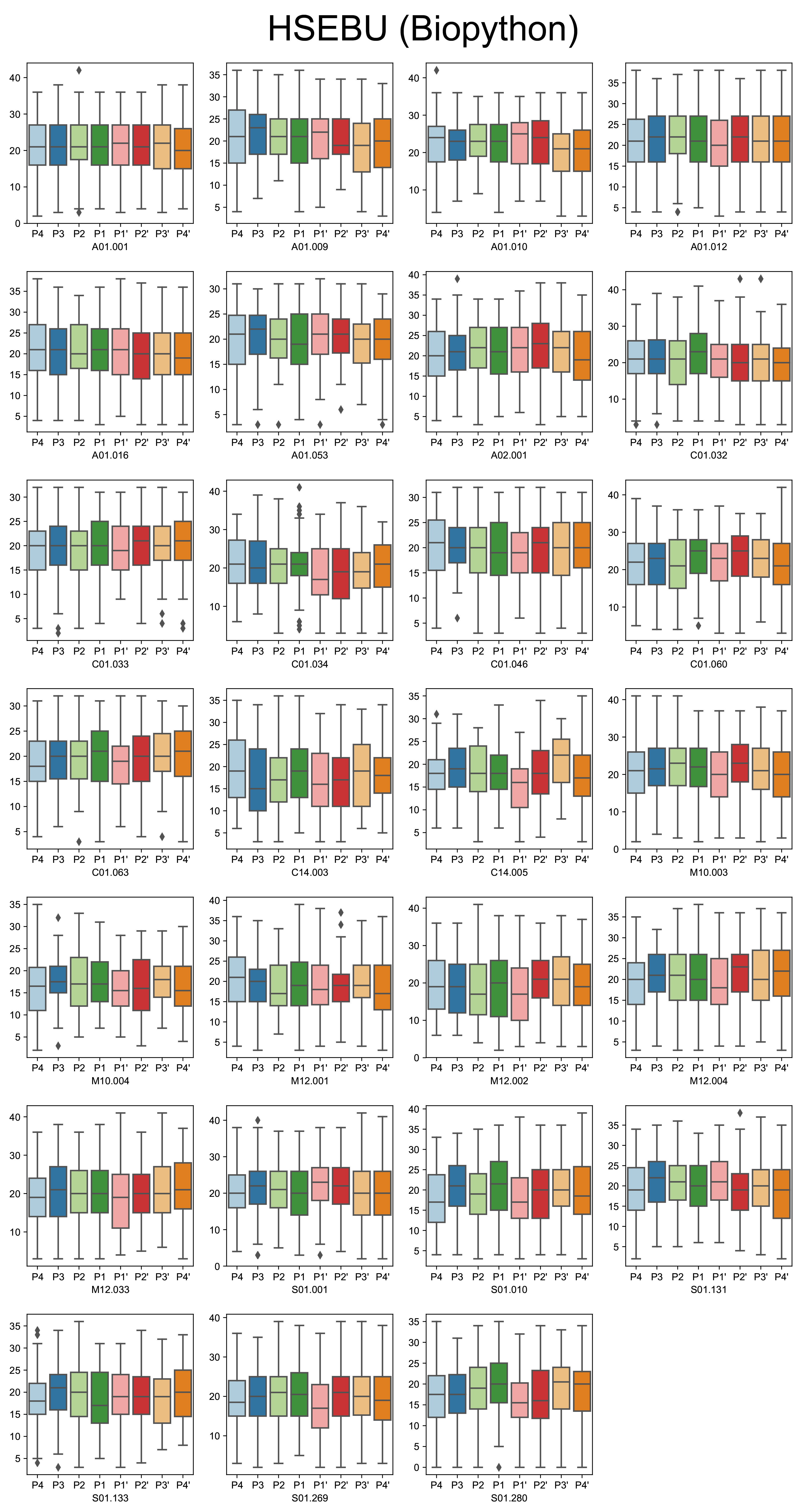

Supplement: Supplementary Figure S23 — Boxplots of HSEBU calculated by Biopython. [file mmc29.zip › Figure S23.png]

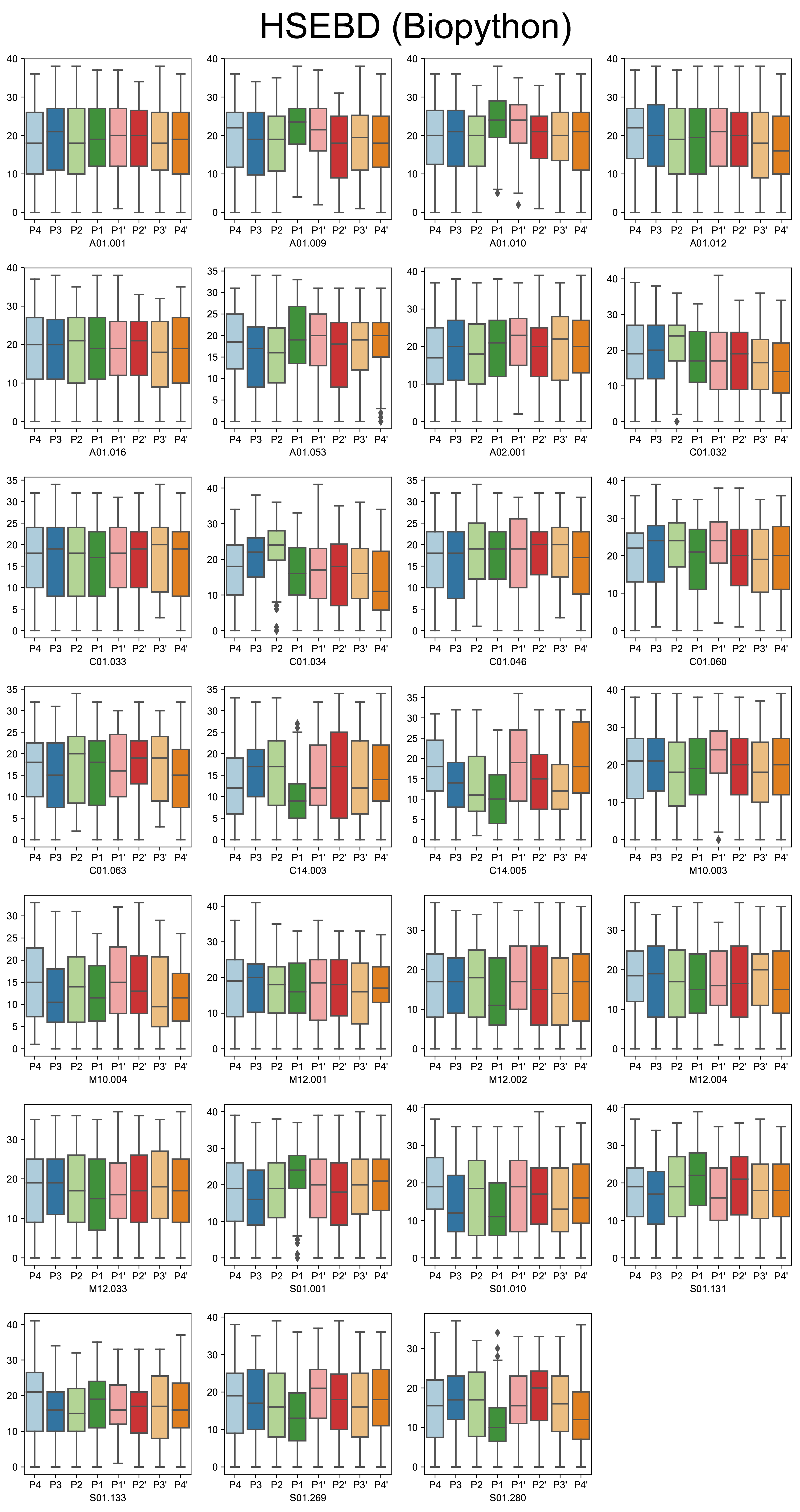

Supplement: Supplementary Figure S24 — Boxplots of HSEBD calculated by Biopython. [file mmc30.zip › Figure S24.png]

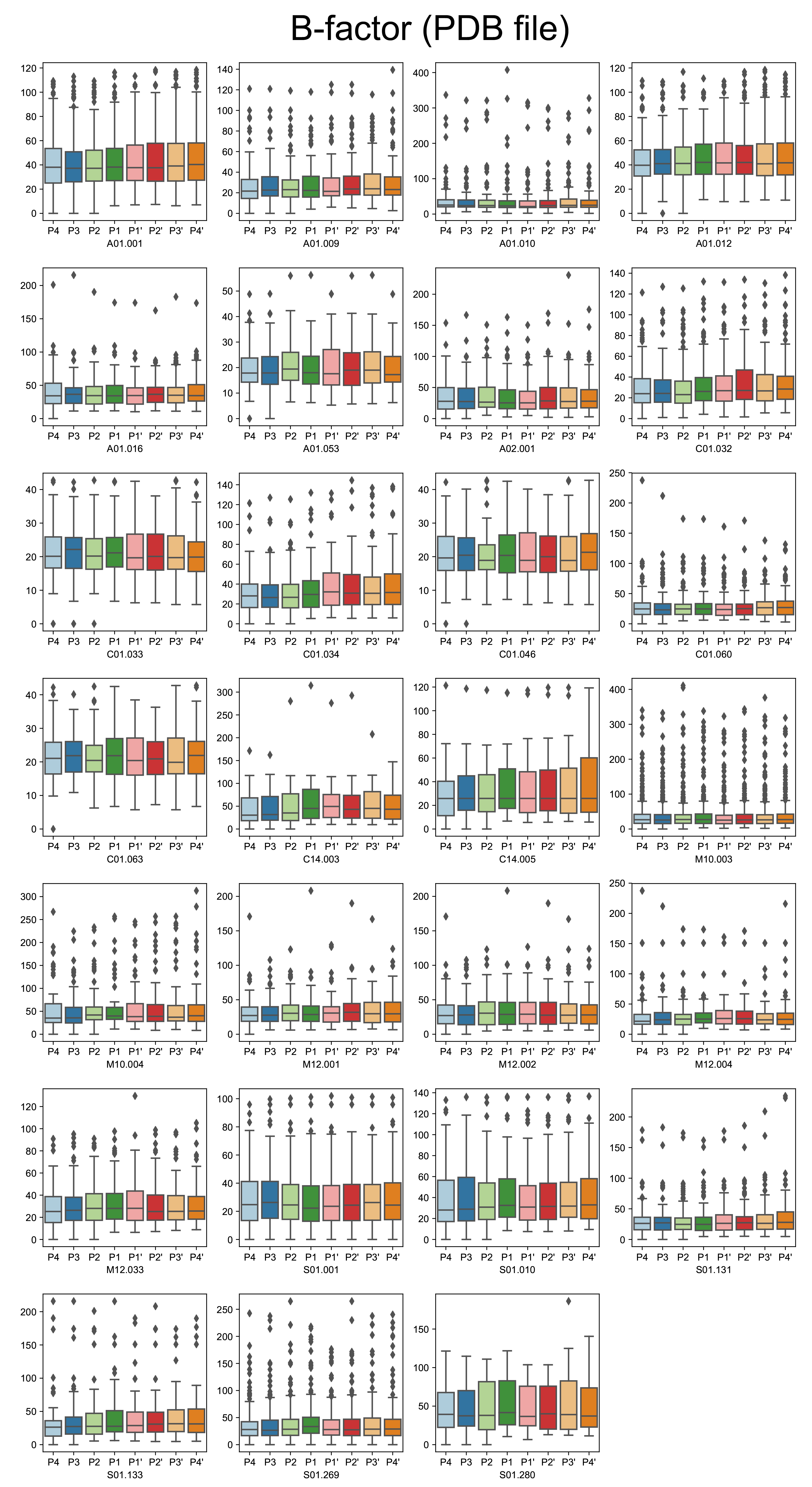

Supplement: Supplementary Figure S25 — Boxplots of B-factor extracted from PDB files. [file mmc31.zip › Figure S25.png]

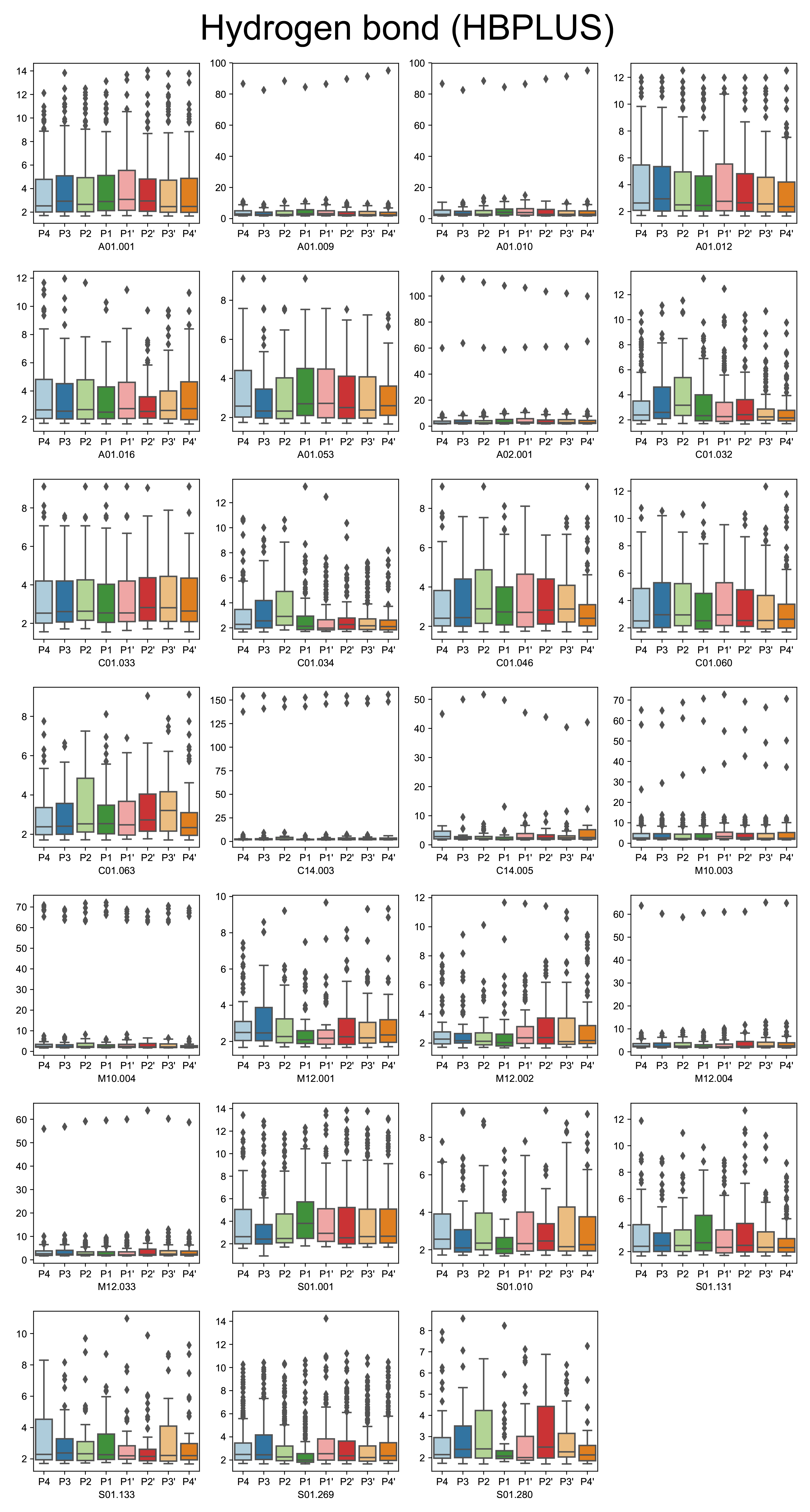

Supplement: Supplementary Figure S26 — Boxplots of hydrogen bond calculated by HBPLUS. [file mmc32.zip › Figure S26.png]
